# Interstitial Nonmetal Doping of Palladium-Based Catalysts for Alkyne Semihydrogenation

**DOI:** 10.1021/acsnanoscienceau.6c00134

**Published:** 2026-09-08

**Authors:** Ruiyun Guo, Yuan Ren, Qixiao Han, Yanjin Wei, Jinyuan Zhang, Can Tang, Yibin Liu, Xiaobo Duan, Anyu Feng, Mingshang Jin

**Affiliations:** † School of Materials Science and Engineering, 118249 Xi’an University of Science and Technology, Xi’an, Shaanxi 710054, China; ‡ Frontier Institute of Science and Technology and State Key Laboratory Multiphase Flow in Power Engineering, 12480 Xi’an Jiaotong University, Xi’an, Shaanxi 710049, China; § Interdisciplinary Research Center of Frontier Science and Technology, Xi’an Jiaotong University, Xi’an, Shaanxi 710049, China

**Keywords:** Pd-based catalysts, interstitial nonmetal doping, alkyne semihydrogenation, electronic effect, geometric effect, thermocatalysis, electrocatalysis, adsorption modulation, scaling relationship

## Abstract

Selective hydrogenation of alkynes over Pd-based catalysts is a core industrial process for purifying crude alkenes from steam cracking and synthesizing high-value-added fine alkene chemicals. Despite extensive research efforts, achieving simultaneous improvement in alkene selectivity and catalytic activity remains a long-standing challenge, as traditional Pd site modulation strategies are inherently limited by the scaling relationships between alkyne and alkene adsorption energies. In recent years, interstitial nonmetal (*e.g.*, B, C, N, P, S) doping has emerged as a promising modulation strategy to simultaneously boost alkyne conversion and alkene selectivity in Pd catalysis. The synergistic electronic and geometric effects induced by interstitial nonmetal atoms modulate the adsorption affinity of reactants and products, enabling Pd to preferentially adsorb alkynes, while simultaneously isolating active Pd ensembles or suppressing subsurface hydrogen formation, thereby enhancing the overall catalytic performance. In this review, we provide the first systematic overview of recent advances in interstitial nonmetal-doped Pd catalysts for selective alkyne hydrogenation, with an elaboration of their intrinsic modulation mechanisms. We begin by clarifying the fundamental mechanisms of Pd-catalyzed alkyne semihydrogenation, the limitations of traditional modification strategies, and the unique merits of interstitial nonmetal doping. Then, we highlight the catalytic performance and underlying modulation mechanisms of different interstitial nonmetal dopants for improving the selectivity of Pd catalysts in both thermocatalytic and electrocatalytic alkyne semihydrogenation. Finally, we outline the existing challenges and future perspectives for the development of high-performance interstitial nonmetal-doped Pd-based catalysts toward alkyne semihydrogenation.

## Graphical abstract

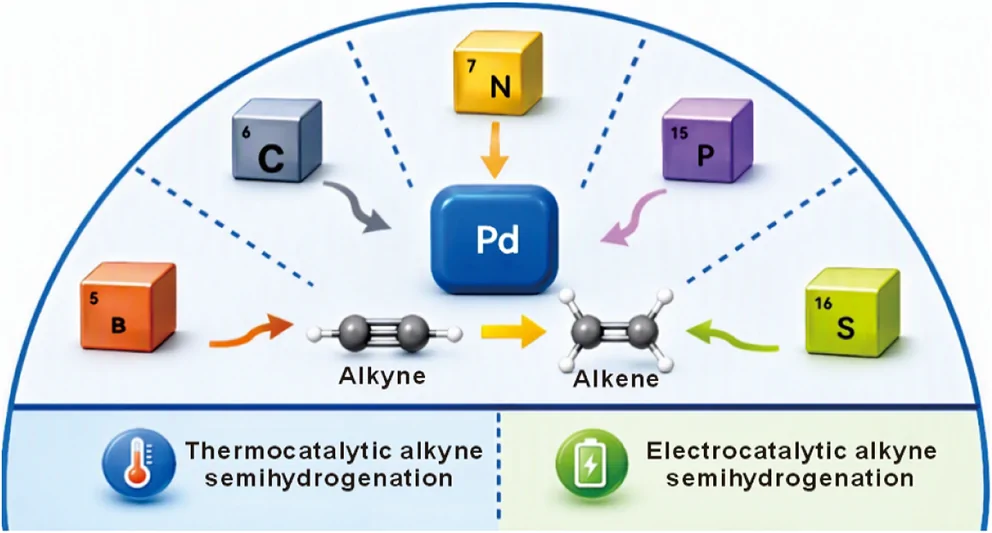

## 1 Introduction

Selective hydrogenation of alkynes to alkenes is a pivotal catalytic process, occupying a crucial position in the chemical industry and also acting as a classic model reaction for investigating catalytic hydrogenation mechanisms.^1,2^ In particular, steam-cracked ethylene contains 0.5–3% acetylene impurities, which poison downstream Ziegler-Natta polymerization catalysts and degrade polyethylene quality.^3,4^ Alkyne semihydrogenation has proven to be one of the most efficient approaches for removing these impurities to below 5 ppm to meet the purity requirement for polymer-grade ethylene.^5^ The hydrogenation of acetylene toward ethylene stands as the most extensively practiced large-scale industrial case of alkyne semihydrogenation, while other light alkynes, including propyne and butyne in C_3_/C_4_ hydrocarbon cuts, are also treated via analogous catalytic routes in refineries.^4^ Besides this application, the selective hydrogenation of liquid-phase substituted alkynes represents an important synthetic route to high-value-added alkenes for fine chemicals, pharmaceuticals, nutraceuticals, and agrochemicals.^6–8^ Representative industrially relevant transformations include the hydrogenation of phenylacetylene to styrene, 2-butyne-1,4-diol to 2-butene-1,4-diol (a vital precursor for vitamin B_6_ and agrochemicals), and various alkynols to alkenols for pharmaceutical and fragrance manufacture.^6–8^ These systems have attracted intensive research attention, owing to both their substantial commercial value and the persistent challenge of attaining high target alkene selectivity while inhibiting over-hydrogenation and cis/trans isomerization side reactions. Meanwhile, from an academic standpoint, this reaction also serves as a classic model system to clarify the structure-activity relationships among active site configuration, surface adsorption characteristics, and catalytic properties.^9,10^ Notably, gas-phase and liquid-phase semihydrogenation impose distinct constraints on catalyst performance. Gas-phase industrial acetylene purification avoids solvent interference and mass-transport limitations, yet high-temperature operation raises risks of metal sintering and oligomerization side-reactions. By contrast, liquid-phase semihydrogenation for fine-chemical synthesis benefits from solvent-mediated surface modulation that can tune intermediate adsorption and improve alkene selectivity, while it suffers from inherent drawbacks including solvent-competitive adsorption, mass-transfer resistance, and potential catalyst leaching. Regardless of reaction phase, however, alkyne semihydrogenation to produce target alkenes inevitably suffers from the detrimental side-reaction of over-hydrogenation, yielding alkanes. These undesirable byproducts reduce reaction efficiency and significantly impede the downstream purification process. Developing robust hydrogenation catalysts with high activity and selectivity is therefore essential to suppress these side pathways and enhance the efficacy of the selective conversion of alkynes to alkenes.

Palladium (Pd)-based metal catalysts are the most widely used materials for alkyne semihydrogenation, owing to their unique electronic structure and strong ability to adsorb and activate hydrogen, which endows them with high intrinsic activity.^11,12^ However, pristine Pd catalysts exhibit excessive adsorption affinity toward alkene intermediates and easily generate subsurface *β*-PdH hydride, often leading to undesirable overhydrogenation toward alkanes, especially at high alkyne conversions.^13–15^ The rational design of Pd nanocatalysts possessing both high activity and selectivity is vital for reducing byproduct formation and has become a major research hotspot in catalysis. Over the past decades, various strategies have been explored to improve the selectivity of Pd catalysts, including poison addition (Pb or quinoline),^16,17^ organic ligand functionalization,^18,19^ and alloying with inert metals.^20–22^ Nevertheless, these approaches suffer from complex modification procedures, an unsatisfactory trade-off between activity and selectivity, and poor tolerance to functional groups, which greatly restrict their practical application. Therefore, it is of great significance to explore alternative efficient modification strategies to enhance the catalytic performance and practicality of Pd catalysts.

Among the considerable efforts devoted to developing Pd-based catalysts for alkyne semihydrogenation, interstitial doping of Pd with nonmetallic atoms (*e.g.*, B, C, N, P, and S) has recently emerged as a promising strategy and received extensive attention due to its remarkable catalytic performance.^23–27^ Studies have shown that such interstitial nonmetal doping can modulate the electronic and geometric structures of Pd, optimizing the adsorption affinity of reactant species on Pd active sites and suppressing *β*-PdH formation or isolating Pd active ensembles, thereby improving catalytic performance in alkyne semihydrogenation.^23–27^ On one hand, the relatively high electronegativity of nonmetals modifies the d-band electronic structure, adjusting the adsorption energy of reaction intermediates and reducing the activation energy, thus affecting both catalytic activity and selectivity. On the other hand, nonmetallic atoms incorporated into the Pd lattices either inhibit the diffusion of subsurface *β*-H and the subsequent formation of PdH_*x*_, which is widely recognized as a major cause of overhydrogenation, or segregate contiguous Pd active ensembles, thereby effectively suppressing excessive hydrogenation side reactions. Collectively, these effects offer an innovative route to address the selectivity bottleneck in alkyne hydrogenation. In recent years, with deepening research on interstitial nonmetal doping strategies and advances in *in-situ* techniques for probing hydrogenation reaction intermediates, remarkable progress has been achieved in nonmetal-doped Pd catalysts for alkyne hydrogenation.^23–32^ These advances have opened up new avenues for the rational design of high-performance Pd-based catalysts with balanced activity and selectivity.

Although encouraging progress has been achieved in nonmetal-doped Pd-based catalysts for the alkyne semihydrogenation, systematic studies focusing on the intrinsic mechanism and specific application underlying the selectivity promotion of Pd catalysts are still lacking. It is therefore highly valuable to systematically review the modulatory roles and applications of interstitial nonmetal doping in alkyne hydrogenation selectivity, which will guide the rational design and industrial implementation of high-performance Pd-based catalysts. For the first time, this review systematically focuses on recent advances in nonmetal-doped Pd-based nanocatalysts for alkyne semihydrogenation (Figure 1). Firstly, we elaborate on the catalytic mechanism of Pd-catalyzed alkyne semihydrogenation, discuss performance improvement strategies, and analyze the unique advantages of nonmetal doping in optimizing the catalytic properties of Pd catalysts. Secondly, we summarize the research progress of interstitial nonmetal (*e.g.,* H, B, C, N, P, S) doped Pd-based catalysts in two current mainstream application fields: thermocatalytic and electrocatalytic semihydrogenation, and clarify the modulation effects and intrinsic mechanisms of different dopants on catalytic performance. Finally, the major challenges confronting interstitial nonmetal-doped Pd catalysts for alkyne semihydrogenation are systematically examined, followed by an outline of their future development prospects. This review aims to provide in-depth insights into the selectivity enhancement mechanism of interstitial nonmetal doping and offer valuable guidance for the fabrication of high-efficiency Pd-based catalysts.

**Figure 1:**
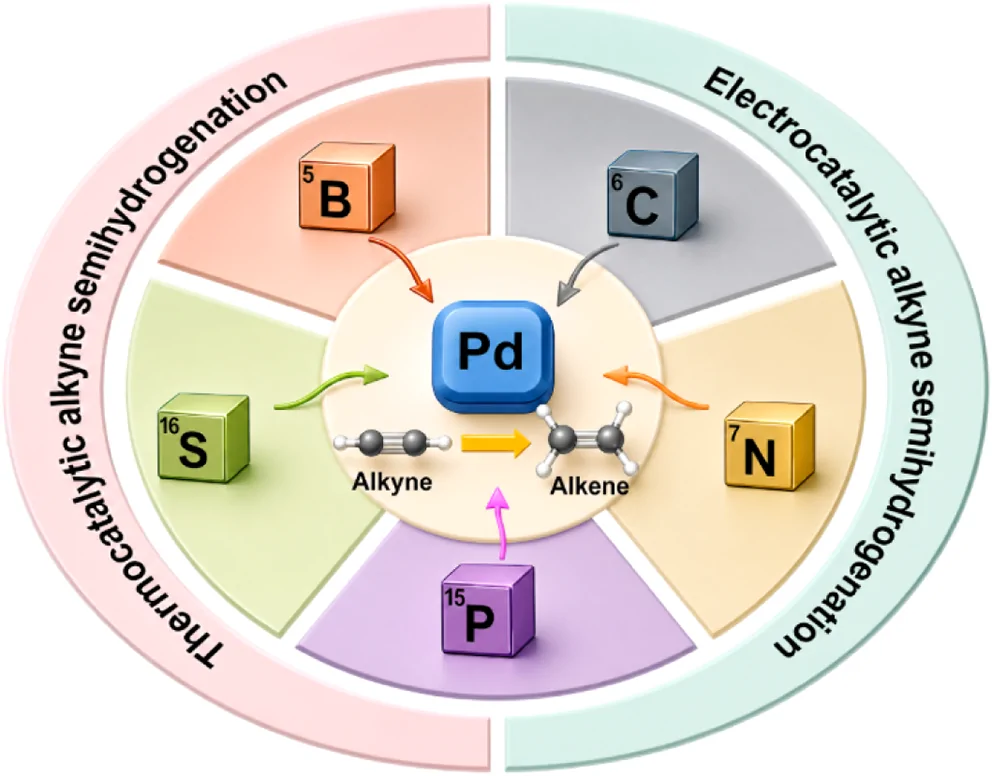
Schematic illustrations of nonmetal-doped Pd-based nanocatalysts for alkyne semihydrogenation.

## 2 Promising Modulation of Pd Catalysts by Nonmetal Doping for Alkyne Semihydrogenation

### 2.1 Fundamental Mechanisms of Pd Catalysts toward Alkyne Semihydrogenation

To clarify the origin of catalytic selectivity in alkyne semihydrogenation, two core selectivity mechanisms, including thermodynamic and kinetic selectivity, are illustrated in Figure 2.^7^ Thermodynamic selectivity originates from competitive adsorption between alkyne and alkene molecules, where alkynes preferentially adsorb on catalyst surfaces (K_1_/K_–1_ ≫ K_3_/K_–3_) and thereby suppress the consecutive overhydrogenation pathway toward alkane byproducts.^33^ In principle, high selectivity requires strong adsorption of alkyne reactants and weak adsorption of as-formed alkenes to facilitate timely alkene desorption. In this regard, adsorption energy serves as a reliable descriptor for correlating catalyst structural features with catalytic selectivity.^1,34^ However, Density functional theory (DFT) calculations by Nørskov *et al.* verified that alkyne and alkene adsorption strengths are linearly correlated due to their analogous surface binding configurations, making the precise regulation of selectivity inherently restrictive.^1^

**Figure 2:**
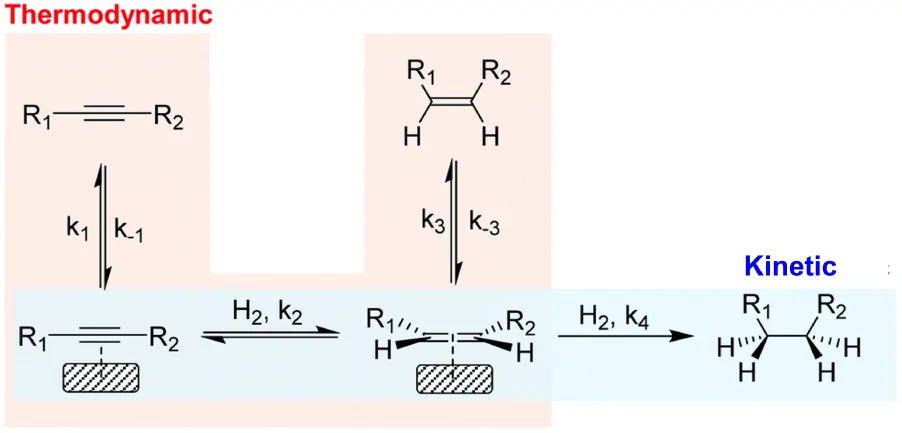
Schematic illustration of alkyne hydrogenation pathways revealing the thermodynamic and kinetic mechanisms. Reproduced with permission from ref^7^. Copyright 2017 Royal Society of Chemistry.

Kinetic selectivity constitutes another vital factor governing catalytic performance, arising from divergent hydrogenation rates of surface-adsorbed intermediates via the classic Langmuir-Hinshelwood mechanism.^35^ Both alkyne hydrogenation to alkene and successive alkene hydrogenation to alkane are limited by surface hydrogenation steps, and high alkene selectivity can be achieved when the hydrogenation rate of adsorbed alkynes far exceeds that of adsorbed alkenes (K_2_ ≫ K_4_).^36^ It is well-established that adsorption thermodynamics and surface reaction kinetics are intrinsically coupled.^7^ Adsorption equilibrium governs the surface coverage of individual reaction intermediates, which in turn shapes the overall apparent hydrogenation kinetics of the system.

Among various catalysts developed for alkyne semihydrogenation, Pd-based materials are the most extensively investigated owing to their outstanding H_2_ dissociation capability and high intrinsic activity. Nevertheless, their strong hydrogen activation ability inevitably facilitates the undesirable overhydrogenation. Coupled with the kinetic-dominated feature of this exothermic reaction under mild conditions, pristine Pd catalysts often suffer from poor alkene selectivity, particularly at high alkyne conversion.^2,37^ As the electronic and geometric structures of Pd simultaneously disrupt favorable adsorption equilibrium and kinetic matching, rational modulation of intrinsic physicochemical properties of Pd is indispensable to balance substrate adsorption, hydrogen activation, and surface reaction kinetics, to boost target alkene selectivity and restrain undesired alkane formation.

### 2.2 Regulation Strategies of Pd Catalysts for Alkyne Semihydrogenation

Extensive literatures have demonstrated that the selectivity of metal catalysts is mainly determined by the electronic effect induced by the metal coordination environment and the geometric effect caused by surface modification.^38,39^ From the perspective of catalytic performance and practical application, the design strategies for high-performance Pd-based alkyne semihydrogenation catalysts can be implemented from the following four aspects: (i) modulating Pd electronic structure to enlarge the adsorption energy difference between alkynes and alkenes; (ii) fragmenting continuous Pd ensembles to mitigate overhydrogenation of the desired alkene products; (iii) tuning surface H* coverage and lattice hydrogen-storage capacity to suppress excessive active hydrogen-induced side reactions; (iv) improving structural stability against nanoparticle sintering and coking to sustain long-term catalytic operation.^11,13,40–42^ On this basis, state-of-the-art strategies to tailor the selectivity of Pd-based nanocatalysts mainly focus on three aspects.

Alloying strategy for geometric isolation and electronic modulation. Breaking Pd ensembles and tuning the binding energies of reaction intermediates are the most direct approaches to elevate semihydrogenation selectivity, which can be effectively realized by alloying Pd with transition metals such as Au, Ag, and Cu, or constructing intermetallic Pd nanomaterials with Zn, Ga, and In. For example, for industrial gas-phase alkyne hydrogenation, Pd–Ag, Pd–Au, or Pd–Cu alloys are the most widely adopted systems to boost alkyne selectivity.^20,43–46^ In such systems, the synergistic regulation of electronic and geometric effects among different metals may modify the binding energies of both alkyne and alkenes, and obtain a better isolation of the active Pd sites, thereby changing the adsorption and desorption behaviors in key reaction steps and ultimately modifying the catalytic selectivity of Pd.
Electronic tuning via strong metal-support interactions. Another strategy involves constructing strong metal-support interactions by employing various supports, such as alumina, carbon materials, graphene, polymers, and metal-organic frameworks.^40^ Besides improving the dispersion of Pd-based catalysts, the electronic transfer or delocalization arising from Fermi-level equilibration between Pd nanoparticles and the supports can effectively modulate the charge density and distribution of Pd active centers, thereby improving the catalytic selectivity of Pd.^47^ It is worth noting that the unique internal environments and defect structures of the supports enable the fabrication of Pd single-atom catalysts, which always display outstanding chemoselectivity because reactants possess only a single adsorption configuration.^2,48^ In thermal catalytic systems, supports also govern the sintering resistance of Pd nanoparticles under high-temperature conditions. Moreover, the surface acid–base properties of supports can further influence secondary side reactions, such as C–C coupling and the formation of green oil or coke.^40^ Additionally, certain reducible oxide supports actively participate in hydrogen spillover, thereby reshaping the hydrogen supply pathways at the metal-support interfaces.^49^
Site passivation via surface modification. Selectively blocking high-activity Pd sites and inhibiting Pd hydride formation are critical to prevent overhydrogenation, which can be implemented by surface functionalization of Pd nanocrystals. This strategy employs organic ligands including amines, thiophenes, thiols, and quinoline to coordinate with surface Pd sites, thereby modulating the adsorption behavior and improving alkyne selectivity.^18,19^ For example, the industrial standard Lindlar catalyst is fabricated by poisoning Pd/CaCO_3_ with quinoline and lead acetate, on which Pb adatoms strongly weaken alkene adsorption and suppress the formation of Pd hydride species, both of which effectively prevent overhydrogenation to alkanes.^50^

Although the efficacy of the above strategies in modulating Pd selectivity has been proven, each approach suffers from inherent limitations. For instance, the synthesis of Pd alloys and intermetallic compounds typically involves complex preparation procedures, and often requires additional noble metals such as Au and Ag or toxic heavy metals such as Pb, which hinders large-scale industrial implementation. For metal-support catalytic systems, performance is highly sensitive to support properties, and achieving atomic-level precision over active site structure substantially increases synthetic complexity. Surface modification strategies rely on site poisoning by organic or inorganic additives, which blocks a large proportion of Pd active sites and inevitably compromises catalytic activity. As a representative example, only 0.02% of Pd sites are estimated to be catalytically active in a commercial 5 wt% Pd Lindlar catalyst.^50^ Collectively, the low atomic utilization efficiency and widespread use of toxic additives highlight the urgent need for alternative rational design strategies. Such strategies aim to develop hydrogenation catalysts that combine high catalytic activity with excellent alkene selectivity. As an unconventional modification pathway, nonmetal doping can simultaneously address multiple desired qualities mentioned above while avoiding partial drawbacks of traditional strategies, which will be systematically elaborated in the following sections.

### 2.3 Merits of Nonmetal Modulation on Pd-Based Catalysts for Alkyne Semihydrogenation

In recent years, nonmetal doping has emerged as a facile and efficient strategy to modulate Pd-based catalysts for alkyne semihydrogenation, attracting considerable research interest.^23–32^ Common nonmetallic dopants, including B, C, N, P, and S, possess the advantages of abundant reserves, low cost, and facile synthesis. The introduction of these nonmetallic heteroatoms can effectively improve the alkene selectivity of Pd-based catalysts, endowing the modified Pd catalysts with promising practical application potential. Unlike metal doping, nonmetallic dopants exhibit unique structural modulation characteristics due to their inherent atomic properties. Specifically, light nonmetallic atoms such as B, C, and N with small atomic radii and strong affinity for Pd are prone to being incorporated into the interstitial sites of Pd crystals, which effectively modulates the 4d electronic configuration and subsurface microstructure of Pd active sites.^51–53^ In comparison, P and S atoms with relatively larger atomic radii can either occupy Pd interstitial sites or substitute partial Pd atoms to form new crystal phases or amorphous structures.^54–57^ Such diversified structural evolution provides abundant avenues for the precise regulation of catalytic properties. The outstanding performance of nonmetal-doped Pd catalysts is primarily governed by the synergistic interplay of electronic and geometric modulation effects.

Electronic effect: The significant electronegativity difference between Pd and nonmetallic atoms triggers intense interfacial charge transfer and polarizes the electronic state of adjacent Pd atoms. Unlike the d-d orbital coupling observed in metal alloy systems, the s and p orbitals of nonmetallic dopants hybridize with the d orbitals of Pd, forming unique s, p-d orbital hybridization structures.^58,59^ This electronic modification optimizes the d-band center of Pd active sites, which reduces the adsorption energy of alkene intermediates and lowers the apparent activation energy of alkyne semihydrogenation, thereby comprehensively improving catalytic activity and alkene selectivity.
Geometric effect: Pristine Pd metal readily absorbs hydrogen atoms, which rapidly migrate to the Pd subsurface and occupy interstitial sites to form *β*-PdH hydrides. During hydrogenation, the reverse diffusion of subsurface hydrides to the catalyst surface leads to high surface hydride coverage, which induces overhydrogenation and severely diminishes alkene selectivity.^60–63^ Interstitial nonmetal dopants fill the native interstitial voids of Pd lattices, which suppresses the formation and diffusion of subsurface hydrides and inhibits the generation of *β*-PdH phases.^60–63^ In addition, larger P and S dopant atoms can partition contiguous Pd active ensembles through lattice distortion, spatially isolating adjacent Pd sites to avoid multi-site adsorption of alkene products and further curb over-hydrogenation side reactions.

Furthermore, catalytic stability is indeed an essential prerequisite for the practical commercial application of Pd-based alkyne semihydrogenation catalysts. For the nonmetal-doped Pd catalysts, theoretically, the introduction of nonmetallic heteroatoms into the interstitial sites of Pd can effectively strengthen metal–metal bonding and stabilize the Pd crystal framework, improving the fundamental structural and chemical stability of the catalyst.^64,65^ Furthermore, the strong electronic interaction between nonmetallic dopants and the Pd matrix significantly suppresses the surface migration, dissolution, and corrosion of Pd active sites, thereby endowing the modified catalysts with excellent long-term cycling durability.^66,67^

### 2.4 Overview of Thermocatalytic and Electrocatalytic Alkyne Semihydrogenation

Catalytic hydrogenation processes are generally categorized into thermocatalytic, electrocatalytic, and photocatalytic pathways based on their reaction driving forces. By contrast, research on Pd-based catalysts for photocatalytic hydrogenation remains far less extensive relative to thermocatalytic and electrocatalytic systems.^68–70^ Accordingly, this review focuses primarily on recent advances in interstitial nonmetal-doped Pd-based catalysts for thermocatalytic and electrocatalytic alkyne semihydrogenation.

Currently, thermocatalytic hydrogenation represents the most widely deployed conventional hydrogenation technology in both industrial manufacturing and laboratory research, owing to its fast reaction kinetics and high production throughput. Alkyne thermocatalytic hydrogenation is generally accepted to follow the Horiuti-Polanyi mechanism, where H_2_ is first adsorbed and dissociated on the catalyst surface, followed by alkyne adsorption and stepwise addition of two hydrogen adatoms to the unsaturated triple bond.^71^ Within this process, the H_2_ dissociation ability of the catalyst is a key factor governing both reaction kinetics and product selectivity. A wide range of catalysts have been developed for selective alkyne hydrogenation, among which Pd-based catalysts exhibit outstanding intrinsic activity, with turnover frequencies (TOFs) ranging from 0.2 to 1.0 s^–1^.^72^ This superior intrinsic activity enables the widespread application of Pd catalysts in thermocatalytic alkyne semihydrogenation, even under mild temperatures and ambient pressure. Nevertheless, Pd catalysts typically suffer from low alkene selectivity, and side reactions including overhydrogenation and oligomerization remain difficult to regulate, especially at high alkyne conversion levels. Accordingly, preserving the high intrinsic activity of Pd while enhancing selectivity toward target alkene products is critical for improving the atom utilization efficiency of Pd and reducing overall production costs. Furthermore, it should be noted that the use of H_2_ atmospheres as the hydrogen source in thermocatalytic semihydrogenation raises concerns over safety and environmental impact due to associated CO_2_ emissions.^7,73,74^

Recently, electrocatalytic alkyne hydrogenation has emerged as a promising alternative approach for alkyne semihydrogenation. Powered by renewable electricity, this approach enables *in situ* generation of active hydrogen species (H*) via electrochemical dissociation of proton donors under ambient conditions, rendering it highly promising for practical deployment.^75–79^ Pioneering work on electrocatalytic alkyne hydrogenation dates back to 1964, when Kemball and coworkers first reported the electrochemical reduction of acetylene to ethylene on Pd electrodes in alkaline media, verifying the feasibility of electrocatalytic alkyne semihydrogenation.^80^ Nevertheless, early electrocatalytic alkyne hydrogenation systems suffered from low current density (<5 mA cm^–2^), unsatisfactory alkene selectivity (<80%), and insufficient alkyne conversion (<90%). Their catalytic performance was far inferior to that of conventional thermocatalytic hydrogenation, which severely hindered further development of the field.^6^ Over the past decades, advances in nanocatalyst synthesis and the development of advanced reactor devices such as flow electrolyzers have driven remarkable improvements in electrocatalytic alkyne hydrogenation. Its performance is now comparable to or even exceeds that of state-of-the-art thermocatalytic systems.^77–79,81–84^ Notably, the use of abundant, safe, and eco-friendly H_2_O as the proton source further improves the sustainability of this approach. It eliminates dependence on high-pressure exogenous H_2_ gas and the associated safety risks of H_2_ storage and transportation. Additionally, its broad substrate scope for diverse alkene synthesis and flexibly tunable operating conditions endow electrocatalytic alkyne hydrogenation with great potential for large-scale production of both bulk and fine chemicals to meet diverse market demands.^85,86^

Overall, from the fundamental mechanism of these two semihydrogenation routes, thermocatalytic semihydrogenation relies on thermally activated H_2_ dissociation over Pd surfaces, where reaction temperature and H_2_ partial pressure are dominant parameters governing surface H* coverage and catalytic behavior. In sharp contrast, electrocatalytic semihydrogenation proceeds via proton-electron transfer pathways, where applied potential modulates interfacial H* population, and local electrolyte environment substantially alters substrate adsorption energetics, rather than thermal input. Despite distinct operating principles, both catalytic platforms are confronted with the persistent challenge of balancing high activity and favorable alkene selectivity, which has stimulated extensive research on modified Pd-based catalysts. In recent years, the significant application value and substantial research progress of interstitial nonmetal-doped Pd-based catalysts in alkyne semihydrogenation have been widely recognized and systematically investigated.^23–32^ This review systematically summarizes recent advances in these modified Pd-based catalysts for both thermocatalytic and electrocatalytic hydrogenation systems. It aims to provide effective strategies and practical insights for addressing the longstanding challenge of precise selectivity control in alkyne semihydrogenation.

## 3 Interstitial Nonmetal Doping of Pd for Thermocatalytic Alkyne Semihydrogenation

As outlined above, the catalytic performance of metallic Pd is governed by its electronic structure and ensemble effects, which are primarily determined by the intrinsic properties of Pd nanoparticles, including particle size, geometric morphology, and initial surface-lattice defects. Collectively, these features modulate the Pd d-band center and substrate adsorption affinity. Specifically, small Pd nanoparticles can deliver abundant active sites with high turnover frequency, whereas alkene-selectivity does not always follow a simple size-dependent trend.^40^ Moreover, initial morphology and exposed crystal facets also alter dynamic doping behaviors under working conditions, thereby reshaping the actual active phase in-situ and catalytic performance.^87^ Meanwhile, surface-lattice defects serve as high-energy sites that can modify the adsorption strength of reactants and intermediates. When incorporated into the Pd lattice, nonmetallic atoms such as B, C, N, P, and S can synergistically regulate the catalytic properties of Pd. On the one hand, they will tune the electronic structure of Pd via charge transfer and downshift its d-band center, fundamentally weakening the excessive adsorption of reaction intermediates. On the other hand, they can effectively block the penetration of hydrogen atoms into the Pd bulk through lattice occupancy and steric hindrance effects to inhibit the enrichment of subsurface hydrogen and *β*-PdH formation, thus achieving simultaneous improvement of activity and selectivity in alkyne semihydrogenation. Inspired by the above modulation mechanisms, targeted development of nonmetal-doped Pd nanocatalysts has therefore emerged as a feasible and attractive route to optimize the performance of alkyne semihydrogenation. Except for N (for which reports are scarce), we systematically review, in the following sections, the research progress of B-, C-, P-, S-, and multi-nonmetal-doped Pd-based catalysts in thermocatalytic semihydrogenation according to the type of doped nonmetallic elements. We focus on the doping modes, structural regulation effects, and catalytic performance enhancement mechanisms of different nonmetallic elements.

### 3.1 Pd–B Catalysts for Alkyne Semihydrogenation

As mentioned above, boron (B) has a small atomic radius and can readily occupy the interstitial lattice sites of metallic Pd. Systematic structural and bonding analysis of nanoscaled metal borides has shown that its moderate electronegativity enables thermodynamically favorable incorporation into Pd octahedral sites via strong host-guest interactions.^88^ This incorporation induces orbital hybridization between Pd d-orbitals and B s-p orbitals, driving electron transfer from interstitial B to Pd. Consequently, the d-electron density and d-band filling of Pd are elevated, altering the electronic and adsorption properties of the Pd-based catalysts.^23,88,89^ Taking advantage of this electronic modulation effect, researchers have achieved the controllable synthesis of Pd–B nanocrystals via diverse synthetic strategies and demonstrated their superior catalytic properties across a wide range of applications.^90–93^ These studies conclude that B incorporation changes the Pd lattice parameters and modulates charge redistribution at the Pd–B interface, thereby creating an optimized electronic configuration that synchronously boosts catalytic activity and selectivity.

In the field of alkyne selective hydrogenation, Tsang *et al.* were the first to report that interstitial B doping endows Pd catalysts with high alkene selectivity.^23^ They prepared B-modified Pd (Pd-^int^B) nanocatalysts by treating preformed Pd nanoparticles with a borane-tetrahydrofuran solution, and demonstrated that the obtained catalysts deliver superior selectivity for the hydrogenation of both terminal and internal alkynes, even for a variety of industrially important and challenging substrates (Figure 3a,b). In Pd-^int^B nanocatalysts, slight lattice expansion tends to upshift and narrow the Pd d-band, whereas the dominant effective overlap of d and p orbitals leads to a downshift and broadening of the d-band (Figure 3c). As evidence, DFT calculations confirm obvious electron accumulation around the subsurface region adjacent to interstitial B atoms (Figure 3d), revealing strong host-guest electronic interactions and the energetic favorability for B atoms occupying Pd octahedral interstitial sites. Quantitatively, the d-band center shifts down from −1.37 eV for pristine Pd to −2.05 eV, and the d-band width is enlarged to 1.56 eV. The overall downshift and broadening of the Pd d-band weaken substrate adsorption on the Pd–B surface, suppressing cis-trans alkene isomerization as well as undesirable overhydrogenation, hence achieving superior cis-selectivity compared to Pd/C and the Lindlar catalyst (Figure 3e,f).

**Figure 3:**
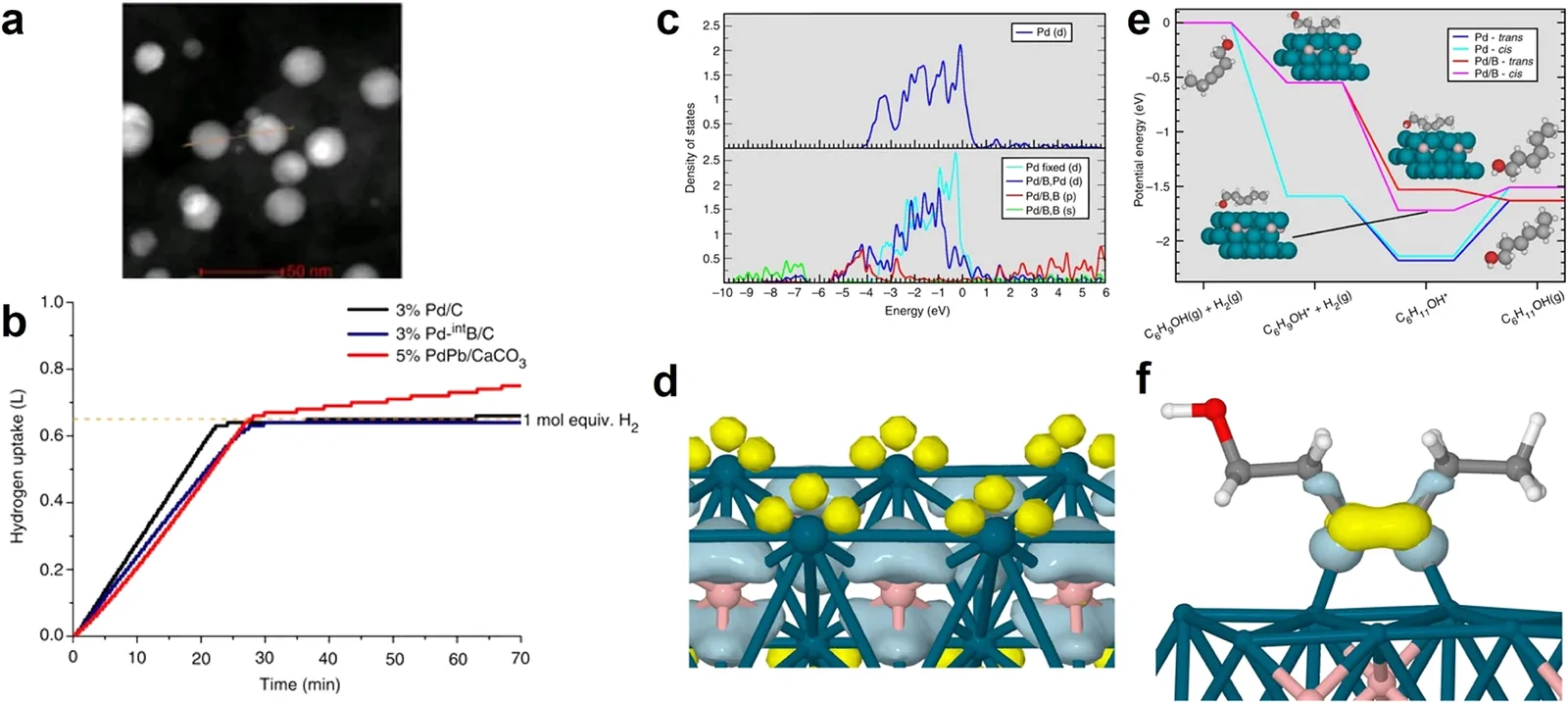
(a) Morphology of the Pd-^int^B nanocatalysts. (b) Hydrogen uptake curves in the hydrogenation of 3-hexyn-1-ol over different catalysts. (c) pDOS of pristine Pd and Pd-^int^B catalysts. (d) Electron density difference for Pd-^int^B (light blue: electron rich, yellow: electron poor). (e) Adsorption energy profiles of 3-hexyn-1-ol on pristine Pd and Pd-^int^B catalysts. (f) HOMO-LUMO isosurface of substrate adsorbed on Pd-^int^B surface. (a–f) Reproduced with permission from ref^23^. Copyright 2014 Springer Nature.

Moreover, benefiting from the modulation of B doping, outstanding chemoselectivity and stereoselectivity toward cis-alkene formation were also achieved over the Pd–B/SiO_2_ catalyst.^94^ Specifically, boron species partially block contiguous Pd surface sites for the chemisorption of alkyne and hydrogen, fragmenting large Pd active ensembles into tiny clusters with merely one or two Pd atoms. Meanwhile, the blocked Pd sites reduce the surface coverage of hydrocarbon intermediates, which shortens the residence time of generated alkenes on the catalyst surface and suppresses their secondary isomerization into trans-(E) isomers, collectively accounting for the superior stereoselectivity. Beyond these surface geometric site-blocking observations reported in supported Pd–B catalysts, Ellis *et al.* conducted a systematic mechanistic investigation to uncover the origins of the B-induced performance enhancement.^95^ Their results further confirmed that, unlike the surface site-blocking effect in conventional alloying systems, this subsurface modification does not cover surface active sites and thus improves the utilization efficiency of Pd atoms. The incorporation of B atoms lowers the Fermi-level electron density on the Pd surface, leading to weaker adsorption of intermediates yet a faster overall hydrogenation rate. More importantly, the embedded B atoms physically hinder the inward diffusion of hydrogen and effectively suppress the formation of *β*-PdH that would otherwise cause overhydrogenation.^96,97^ As a result of this synergy between structural advantage and electronic regulation, the Pd–B catalysts deliver a consistently high ethylene selectivity exceeding 80% during long-term steady-state reaction, while that of unmodified Pd is only 50%.^97^

Collectively, the above works establish interstitial B doping as a highly effective strategy for enhancing the selectivity of Pd catalysts in alkyne semihydrogenation. The incorporation of B lowers the adsorption strength of the alkene intermediate while suppressing the formation of *β*-PdH, the major cause of overhydrogenation. However, compared with the extensive research on Pd–B catalysts for electrocatalytic applications such as formic acid decomposition, ethanol oxidation, and oxygen reduction, studies on Pd–B catalysts for alkyne semihydrogenation remain relatively scarce. To date, only a limited number of relevant reports have been published in this area. Therefore, more efforts are still required, not only to develop efficient synthetic approaches for high-performance Pd–B catalysts, but also to establish structure-performance correlations and further expand their application in alkyne semihydrogenation.

### 3.2 Pd–C Nanocatalysts for Alkyne Semihydrogenation

Among various nonmetallic modifiers for Pd-based alkyne semihydrogenation catalysts, interstitial carbon (C) doping is by far the most widely investigated strategy for regulating their catalytic performance.^87,98–102^ Conventionally, carbonaceous species generated *via in situ* decomposition of adsorbed carbon-containing substrates during hydrogenation have long been regarded as detrimental contaminants that block active sites and cause gradual catalyst deactivation over time on stream. However, recent computational and experimental studies have overturned this long-held perception, demonstrating that C atoms incorporated into Pd interstitial sites act as a selectivity promoter for alkyne semihydrogenation.^60,103^ The mechanistic understanding of this promotion effect was first advanced by theoretical calculations. Nørskov and coworkers revealed via DFT simulations that subsurface carbon weakens the adsorption of CH_3_ fragments and other carbonaceous intermediates, including alkenes, which accounts for the improved selectivity observed experimentally over subsurface carbon-modified Pd catalysts.^34^ Complementing these theoretical findings, Teschner and coworkers provided direct experimental evidence using *in situ* X-ray photoelectron spectroscopy (XPS) under reaction conditions. They demonstrated that less carbon dissolves in the Pd lattice during unselective total hydrogenation, whereas a significant amount of carbon remains in the lattice under selective semihydrogenation conditions (Figure 4a,b).^60^ Meanwhile, *in situ* prompt gamma activation analysis (PGAA) measurements of hydrogen content confirmed that high-concentration dissolved carbon atoms exclude hydrogen from occupying the subsurface region, thus preventing alkyne overhydrogenation. Further investigations have elucidated the structural origin of this promotion effect, focusing on the formation and dynamic evolution of palladium carbide (PdC*_x_*) phases under reaction conditions. Multiple *in situ* techniques including X-ray diffraction (XRD), X-ray absorption near-edge structure (XANES), and extended X-ray absorption fine structure (EXAFS) have verified that carbonization initiates on the surface of Pd nanoparticles and gradually propagates into the bulk, eventually forming homogeneous PdC*_x_* across the entire particle in the early stage of hydrogenation.^63,104–107^ The formation of PdC_*x*_ modulates both the crystal structures and electronic properties of Pd, and the selectivity enhancement follows two synergistic mechanisms. On one hand, orbital hybridization between Pd d-orbitals and C s-p orbitals tunes the hydrogen activation capability of Pd sites and significantly weakens the adsorption of the target alkene products, thereby improving alkene selectivity. On the other hand, interstitial C atoms strongly hinder the participation of subsurface hydrogen in the reaction and suppress the formation of the undesirable *β*-PdH during catalysis, which is one of the primary causes of overhydrogenation.

**Figure 4:**
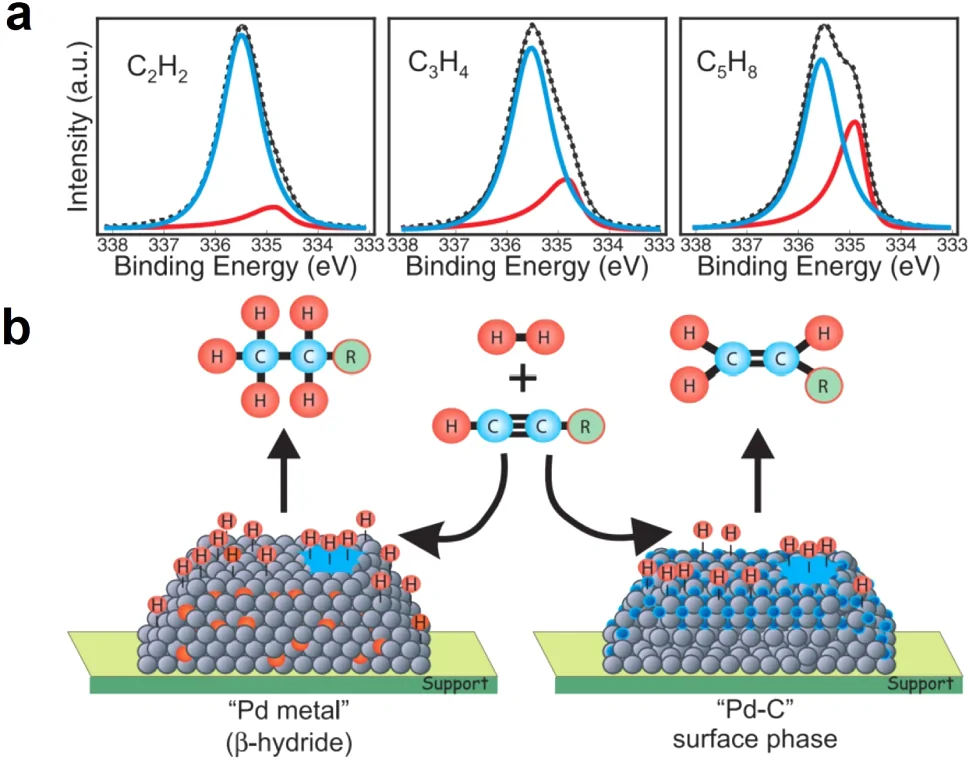
(a) *In-situ* Pd 3d_5/2_ XPS spectra collected during the hydrogenation of acetylene, propyne, and 1-pentyne (red curve: metallic Pd; blue curve: sum of adsorbate-shifted Pd and PdC; dashed line: experimental spectra). (b) Schematic representation of Pd catalysts operating in the selective and unselective alkyne hydrogenation regimes. (a, b) Reproduced with permission from ref^30^. Copyright 2008 American Association for the Advancement of Science.

Despite its effectiveness in selectivity enhancement, the PdC*_x_* phase spontaneously formed during hydrogenation exists only transiently and in low content under reactive gas atmospheres, making it difficult to isolate and use as an independent catalyst. To address this limitation, researchers have developed strategies for the direct synthesis of stable PdC*_x_* nanocatalysts. Tsang *et al.* first fabricated PdC_*x*_ nanocatalysts via glucose pyrolysis, in which the as-formed carbonaceous layer rapidly encapsulates individual Pd nanoparticles and enables interstitial carbon filling during heat treatment.^97,108^ Catalytic tests on the hydrogenation of 3-hexyn-1-ol and 4-octyne confirmed that the obtained PdC_*x*_ catalysts effectively suppress undesired isomerization and overhydrogenation, delivering higher alkene selectivity. However, this approach requires a complex hydrogen peroxide treatment to remove surface carbon for subsequent catalytic reactions, which inevitably reduces the interstitial carbon content. Moreover, this synthetic strategy cannot achieve precise tuning of the carbon content in the as-prepared PdC_*x*_ nanocrystals, which severely limits further quantitative exploration of the correlation between carbon content and catalytic performance. Furthermore, catalytic performance is sensitive to heteroatom doping magnitudes, where both insufficient and excessive doping can modify catalytic behaviors, and the favorable doping range is condition-dependent. To establish a robust structure-performance relationship, considerable efforts have been devoted to the controllable synthesis of PdC*_x_* nanocatalysts with tunable C/Pd atomic ratios. In 2019, our group first reported the synthesis of PdC_*x*_ nanocrystals with precisely tunable carbon content via hydrothermal decomposition of glucose, where carbon atoms are incorporated into the Pd lattice in a controlled manner (Figure 5a).^24^ Corresponding XRD patterns clearly show that the C/Pd atomic ratios in the as-prepared PdC_*x*_ nanocrystals can be tuned from 0.04 to 0.18 simply by adjusting the reaction time (Figure 5b). Notably, catalytic tests demonstrate that interstitial C incorporation effectively boosts the catalytic performance of Pd catalysts. As shown in Figure 5c, the TOFs of PdC_*x*_ catalysts increase with rising C content, and PdC_0.18_ nanocatalysts exhibit an ultrahigh TOF of 7896 h^–1^ for the semihydrogenation of 4-ethynyl-1,1′-biphenyl. This value is 7.6 and 38 times higher than those of commercial Pd/C and Lindlar catalysts, respectively. Regarding the enhancement mechanism, interstitial C atoms hybridize with Pd d bands to alter the Fermi level of the Pd sites and induce preferential adsorption of alkynes, while simultaneously suppressing *β*-PdH formation by hindering subsurface hydrogen incorporation. These two effects collectively endow PdC_*x*_ nanocrystals with excellent selectivity. In addition to the hydrothermal approach, another strategy for precise C content control has been developed via an *in-situ* reduction-carburization process by tuning the hydrogenation atmosphere and employing ZnO additives. A linear correlation between carbon content and acetylene semihydrogenation activity was observed in this system (Figure 5d),^109^ further verifying the composition-dependent performance.

**Figure 5:**
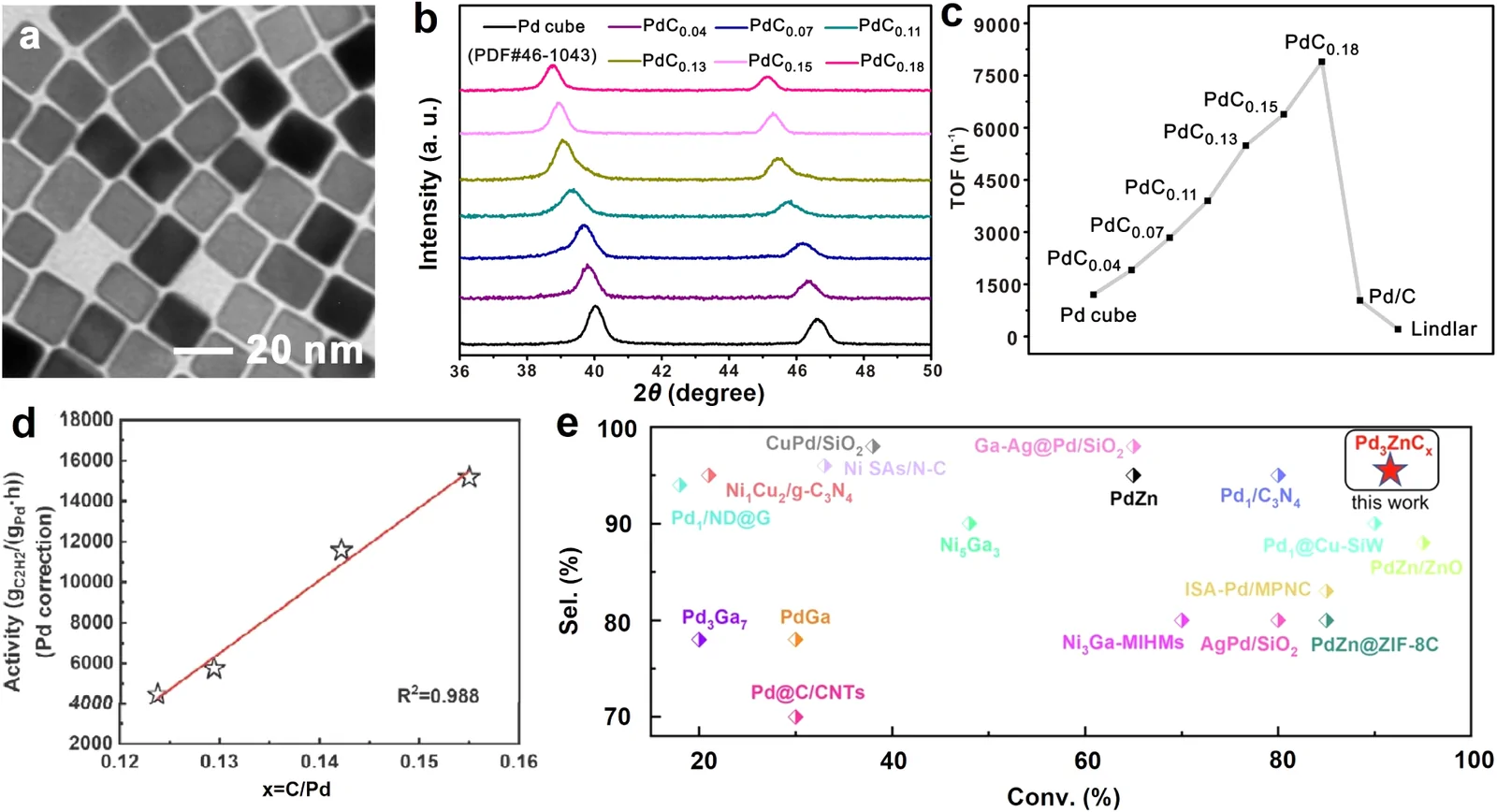
(a) TEM image of the representative PdC_0.18_ nanocatalysts. (b) XRD patterns of PdC*_x_* nanocrystals with C/Pd atomic ratios of 0.04, 0.07, 0.11, 0.13, 0.15, and 0.18. (c) TOF values for the semihydrogenation of 4-ethynyl-1,1-biphenyl over different catalysts. (a–c) Reproduced with permission from ref^24^. Copyright 2019 Royal Society of Chemistry. (d) The linear relationship between mass activity of acetylene semihydrogenation and carbon content of PdC*_x_* catalysts. Reproduced with permission from ref^110^. Copyright 2024 Elsevier. (e) Catalytic performance of Pd_3_ZnC*_x_* and other reported transition metal catalysts in the literature for the semihydrogenation of acetylene. Reproduced with permission from ref^111^. Copyright 2024 Springer Nature.

Encouraged by the unique promotion effect of interstitial C modification, researchers have extended this concept beyond pure Pd systems to further explore its catalytic potential. A notable example is the Pd_3_ZnC_*x*_ bimetallic carbide, which exhibits remarkable performance in acetylene semihydrogenation. It maintains over 90% ethylene selectivity even at a high H_2_/C_2_H_2_ ratio, surpassing the performance of a wide variety of transition metal catalysts and representing the state-of-the-art for this reaction (Figure 5e).^110^ In addition, anchoring PdC*_x_* on functional substrates is another effective approach to further enhance catalytic performance. For instance, PdC*_x_* subnanoclusters confined within silicate MFI zeolites deliver excellent results under mild reaction conditions, achieving 100% conversion of phenylacetylene and 99% selectivity to styrene.^111^ More importantly, this interstitial C modulation strategy has also been successfully extended to Pt-based catalysts, achieving excellent performance in acetylene hydrogenation and demonstrating its universality.^112^

In conclusion, doping interstitial C atoms into the Pd lattice serves as a powerful strategy to tailor catalytic performance by simultaneously modifying the surface geometric and electronic properties of the catalyst. Given the critical roles of metal carbides in alkyne semihydrogenation, their controllable synthesis and mechanism exploration will attract increasing attention, paving the way for the rational design of high-efficiency hydrogenation catalysts in the future.

### 3.3 Pd–P Nanocatalysts for Alkyne Semihydrogenation

Compared with B, C, and N atoms, P and S have relatively larger atomic radii, making them less likely to intercalate into Pd lattices as interstitial dopants. More often, they tend to alter the crystal symmetry of Pd and form stoichiometric Pd phosphides or sulfides.^113–115^ Extensive studies have confirmed that the electronic interaction between P/S and Pd effectively modulates the adsorption strength of alkynyl and alkenyl groups, thus enhancing alkene selectivity in alkyne semihydrogenation.^26,116–124^ Meanwhile, the site isolation effect induced by P/S doping reduces the size of Pd active ensembles and restricts the migration of organic intermediates, which simultaneously suppresses alkene overhydrogenation, C–C coupling, and oligomerization side reactions.^26,116–120,122–124^

For P-modified Pd systems, tuning the P content and crystal phase enables flexible regulation of the physicochemical properties of Pd. To date, a variety of palladium phosphides, including Pd_6_P, Pd_3_P, PdP_2_, Pd_5_P_2_ and amorphous PdP, have been synthesized and employed as efficient catalysts.^109,125–127^ Among them, Zhao and coworkers were the first to apply monodispersed PdP nanoparticles to alkyne selective hydrogenation.^128^ Using triphenylphosphine as the P source, they prepared small-sized Pd_3_P_0.95_ nanocrystals that exhibited outstanding activity and stereoselectivity (Figure 6a and b). In the semihydrogenation of phenylacetylene, the catalyst achieved an alkene selectivity as high as 99%, outperforming commercial Pd/C and Lindlar catalysts in both activity and selectivity. Mechanistic studies revealed that electron transfer from Pd to P generates electron-deficient Pd species, while P atoms partition contiguous Pd sites and hinder the formation of *β*-PdH phase. Such combined effects underlie enhanced catalytic performance. Building on this work, subsequent studies further explored the structure-performance relationship of Pd phosphides with different crystal phases. Anderson *et al.* prepared TiO_2_-supported Pd_3_P and PdP_2_ nanoparticles via an impregnation method.^26^ At full acetylene conversion, PdP_2_ delivered an ethylene selectivity of 84%, markedly higher than the 70% achieved over Pd_3_P (Figure 6c). Combined XPS and CO-IR characterizations confirmed that higher P content further divides continuous Pd sites and forms more isolated active centers. This structural optimization modulates the adsorption and desorption behaviors of reactants and products, ultimately leading to improved selectivity.

**Figure 6:**
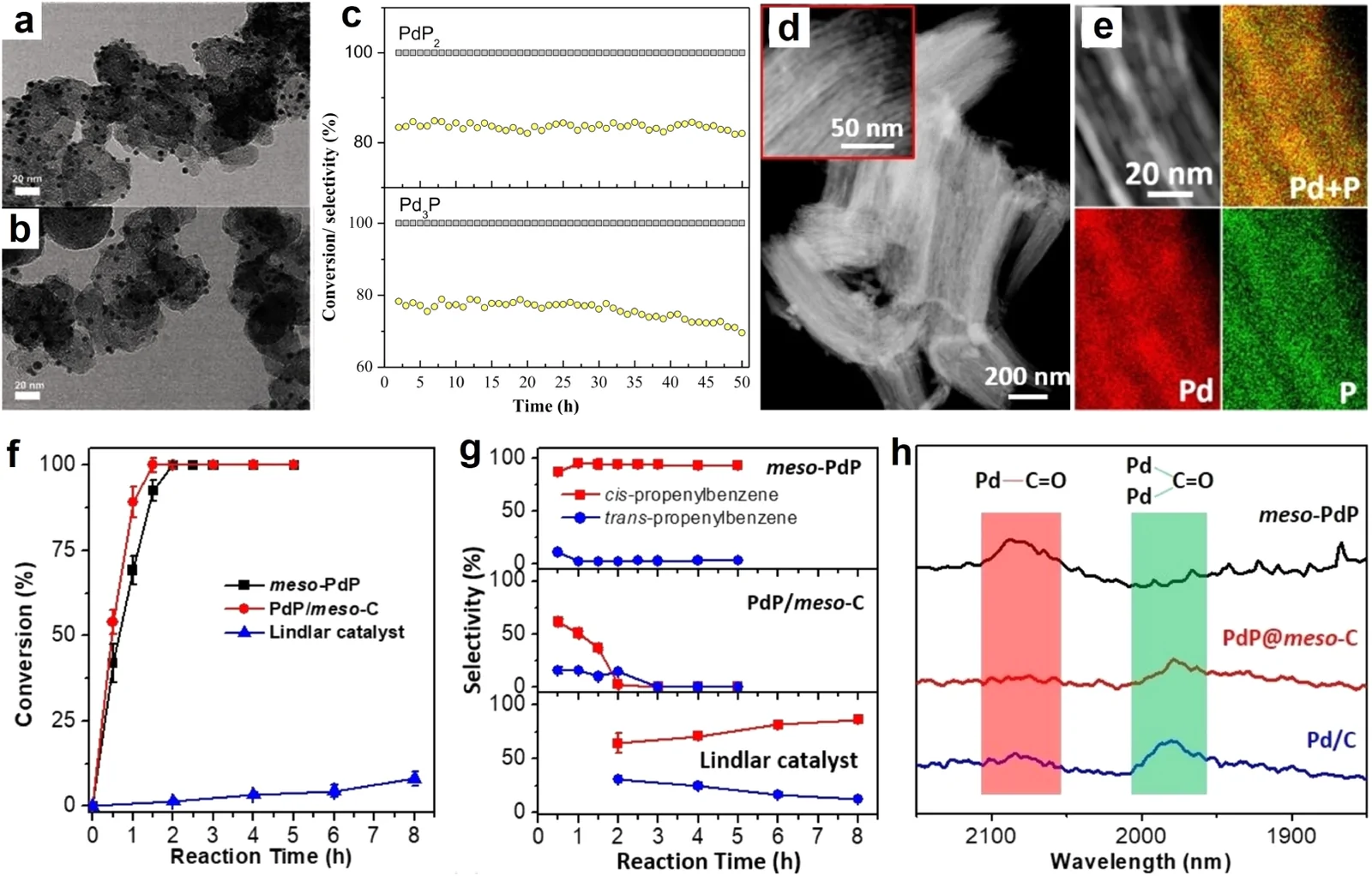
(a, b) TEM images of the obtained Pd_3_P_0.95_/C catalysts. Reproduced with permission from ref^129^. Copyright 2016 Wiley-VCH. (c) Comparison of acetylene conversion (grey squares) and ethylene selectivity (yellow circles) over2% Pd_3_P/TiO_2_ and 2% PdP_2_/TiO_2_. (a–c) Reproduced with permission from ref^26^. Copyright 2018 Elsevier. (d, e) HAADF-STEM and STEM-EDS images of meso-PdP nanobundles. (f) Catalytic activity and (g) selectivity in the hydrogenation of 1-phenyl-1-propyne over meso-PdP, PdP/meso-C, and Lindlar catalysts. (h) CO–DRIFTS over different catalysts. (d–h) Reproduced with permission from ref^122^. Copyright 2022 Wiley-VCH.

Beyond crystal phase and P content tuning, rational structural design and support engineering have become another effective route to further boost the performance of Pd–P catalysts.^120,125^ As a representative example, Bi *et al.* fabricated ultrafine Pd_*x*_P_*y*_ nanoparticles (<2 nm) on defect-rich TiO_2_ via a defect-assisted anchoring strategy.^129^ The surface defects of the support effectively suppressed the aggregation of Pd_*x*_P_*y*_ particles during high-temperature phosphidation. The obtained Pd_*x*_P_*y*_/d-TiO_2_ catalyst showed excellent performance in the selective hydrogenation of furfural to furfuryl alcohol, with a high TOF of 833.8 h^–1^ and 97.5% product selectivity. Here, P doping transforms Pd into isolated and electron-deficient active sites, which weakens the adsorption of furfural and inhibits hydrogen spillover, thereby suppressing various side reactions. In addition to supported nanocatalysts, constructing integrated mesoporous PdP materials is also a promising design strategy for alkyne hydrogenation. Liu group synthesized continuous mesoporous PdP (meso-PdP) nanobundles with penetrating mesochannels (Figure 6d,e).^121^ Unlike isolated PdP nanoparticles, the interconnected crystalline framework of meso-PdP endows the catalyst with unique surface electronic states and adsorption properties. In the semihydrogenation of 1-phenyl-1-propyne, this catalyst achieved a high catalytic activity of 23.4 h^–1^, 97.9% alkene selectivity, and good durability and cycling stability, all superior to meso-Pd, commercial Pd/C, and the Lindlar catalyst (Figure 6f,g). Moreover, it delivered high selectivity for the semihydrogenation of a broad range of alkynes to targeted alkenes, with values exceeding 90%. Combined with XPS, CO diffuse reflectance infrared Fourier transform spectroscopy (CO–DRIFTS), and DFT calculations, the authors attributed the high selectivity to the continuous crystalline framework and interconnected mesoporous channels of the catalysts, which weakened the adsorption affinity and interaction strength between catalysts and alkenes, thereby suppressing overhydrogenation into alkanes (Figure 6h).

Overall, P doping is an effective strategy to engineer Pd-based hydrogenation catalysts. By adjusting P content, crystal phase, particle size, and microstructure, Pd phosphides can be finely tailored for selective alkyne hydrogenation. The synergistic electronic regulation and site isolation effects are widely responsible for their outstanding catalytic performance, and the corresponding design principles offer general guidance for constructing advanced hydrogenation catalysts. Despite the remarkable progress achieved, current studies still have limitations, such as insufficient exploration of long-term stability, poisoning resistance, and practical application potential. Therefore, future work should focus on further optimizing Pd–P catalytic systems, exploring novel structures and clarifying in-depth reaction mechanisms to promote their practical application in alkyne semihydrogenation.

### 3.4 Pd–S Nanocatalysts for Alkyne Semihydrogenation

Analogous to P-modified Pd catalysts, incorporating S into Pd to form Pd sulfides not only significantly alters the crystal lattice structure of bulk Pd, but also modulates its catalytic properties through geometric site isolation and electronic structure regulation.^49,113–115^ Indeed, transition metal sulfides have long been widely utilized in refinery hydroprocessing for their excellent poisoning resistance and hydrogen activation capability, laying a solid practical foundation for the application of Pd sulfide catalysts in selective hydrogenation.^122,123^ Early research on crystalline Pd sulfides for alkyne semihydrogenation focused primarily on the Pd_4_S phase, which shows promising application potential for the hydrogenation purification of trace light alkyne impurities in petroleum-derived alkene streams under gas-phase conditions.^124,130,131^ For instance, supported nano-Pd_4_S catalysts have been extensively validated to be compatible with industrial gas-phase continuous flow hydrogenation conditions. They maintain stable selectivity and activity during long-term operation, and exhibit a much lower coking deactivation tendency than traditional Pd-based catalysts.^132^

Building on these early explorations, growing research efforts have been devoted to constructing Pd sulfide nanocatalysts with diversified phases and structures in recent years, to expand their application scope and improve hydrogenation performance for organic substrates.^27,124,131,133^ Among these studies, the work by Albani *et al.* represents a key advance, providing systematic molecular-level elucidation of the catalytic mechanism of Pd sulfides.^27^ Using sodium sulfide as the S source, they synthesized small-sized Pd_3_S nanocatalysts supported on graphitic carbon nitride via a mild hydrothermal sulfidation method, which enables precise control over the crystalline phase and preferred crystal facet orientation (Figure 7a,b). In the continuous-flow three-phase semihydrogenation of 2-methyl-3-butyn-2-ol, this Pd_3_S/C_3_N_4_ catalyst exhibits unparalleled performance over state-of-the-art reference materials, including classic Lindlar-type PdPb/CaCO_3_, ligand-modified Pd-HHDMA, single-atom Pd@C_3_N_4_, and Pd_4_S-based catalysts (Figure 7c,d). Remarkably, it achieves nearly 100% alkene selectivity simultaneously with a reaction rate 3.5 times that of the Lindlar catalyst under identical conditions. The outstanding performance is attributed to a unique bifunctional surface mechanism, in which H atoms are stored by binding to surface S atoms, while organic reactants are specifically adsorbed on Pd active sites. This eliminates the adsorption competition between alkynes and H on Pd centers, resulting in significantly higher catalytic activity than pure Pd systems. Meanwhile, isolated Pd_3_ surrounded by S atoms exhibits weak adsorption toward reaction intermediates, ensuring rapid desorption of the produced alkenes and kinetically suppressing overhydrogenation.

**Figure 7:**
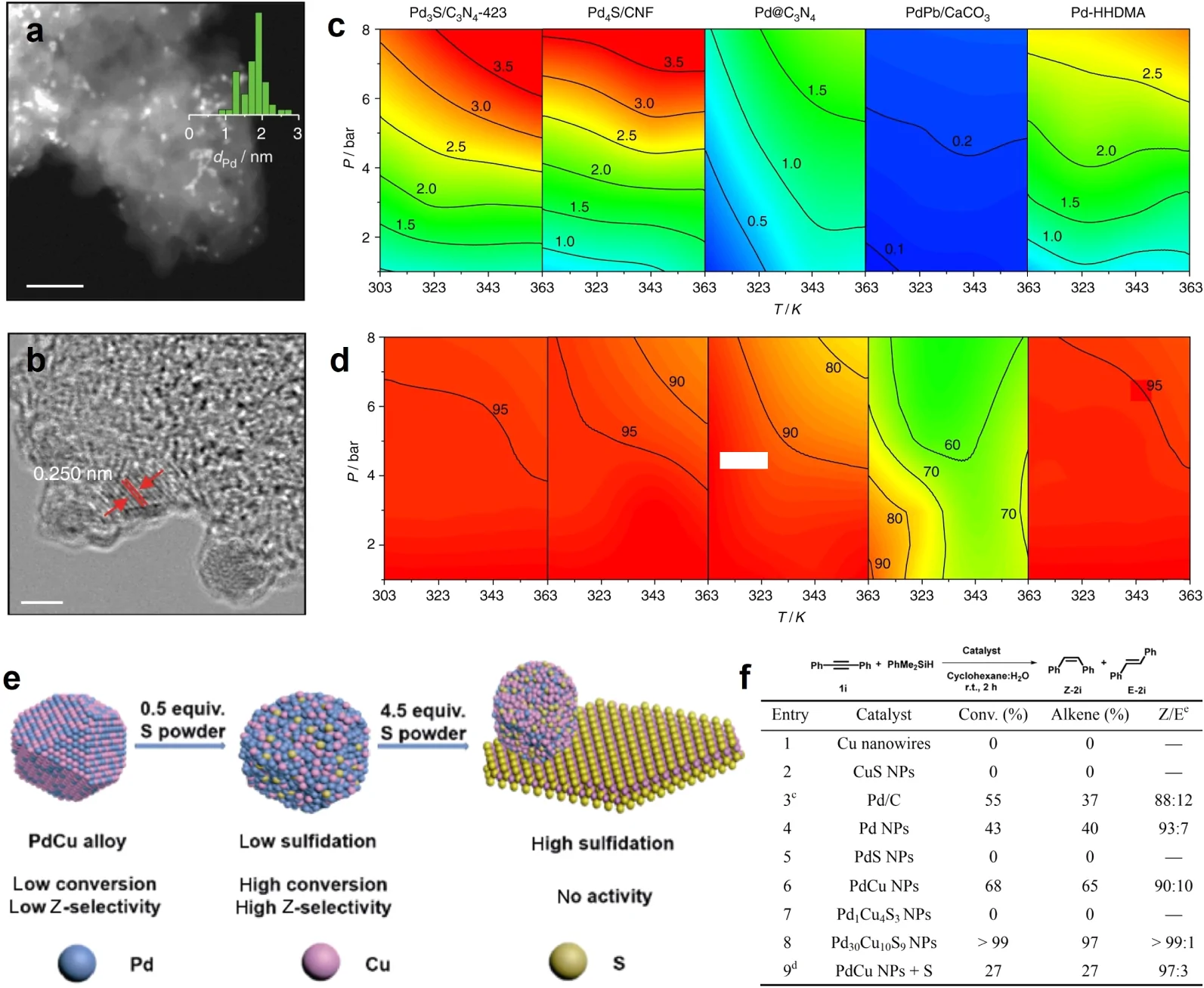
(a) HAADF-STEM image with the particle size distribution (inset) of the Pd_3_S/C_3_N_4_ catalyst, (b) TEM image of the Pd_3_S/C_3_N_4_ catalyst. (c) Contour plot map shows the effect of temperature and pressure on the reaction rate for alkyne semihydrogenation. (d) Selectivity toward 2-methyl-3-buten-2-ol of Pd_3_S/C_3_N_4_ compared with other catalysts. (a–d) Reprinted or adapted with permission under a Creative Commons CC BY license from ref^27^. Copyright 2018 Springer Nature. (e) Promoting effect of low-level sulfidation. (f) Comparison of different catalysts in the 1,2-diphenylethyne semihydrogenation reaction. (e, f) Reproduced with permission from ref^136^. Copyright 2018 Tsinghua University Press and Springer-Verlag GmbH Germany.

In addition to Pd_3_S and Pd_4_S, Pd sulfides with multiple crystalline phases such as PdS and Pd_16_S_7_ have also been applied in alkyne semihydrogenation research, further verifying the universality of the sulfidation modification strategy.^124,131^ To further expand PdS-based catalytic systems and boost catalytic performance, nanostructure engineering and multi-component alloying have emerged as two core optimization directions beyond phase regulation.^134,135^ As a representative example, Liu and coworkers prepared hollow mesoporous PdS (h-mesoPdS) nanoparticles using mesoporous Pd/KIT-6 as a sacrificial template.^134^ This catalyst achieves 94% product selectivity in the selective semihydrogenation of *p*-nitrophenylacetylene to *p*-nitrostyrene, significantly outperforming commercial Pd/C catalysts. DFT calculations further reveal that the crystalline mesoporous structure of h-mesoPdS promotes preferential hydrogenation on the alkyl chain of alkynes. Meanwhile, S incorporation weakens the hydrogen adsorption capacity of Pd sites compared with pure mesoporous Pd, preventing overhydrogenation of the product alkenes and ultimately achieving high selectivity toward *p*-nitrostyrene. Beyond binary Pd–S systems, incorporating S into Pd-based multi-component alloy catalysts can also effectively tune the electronic and geometric properties of Pd active sites. A typical example is the Pd_30_Cu_10_S_9_ nanoparticles synthesized by Wu *et al.*, which exhibit significantly improved activity and selectivity in alkyne semihydrogenation compared with both the unsulfided precursor catalyst and monometallic Pd sulfide reference catalysts.^135^ Nevertheless, the sulfidation degree imposes a critical threshold effect. Excessive sulfidation poisons catalytic active sites, and the as-formed Pd_1_Cu_4_S_3_ virtually loses hydrogenation activity entirely (Figure 7e,f).

Collectively, S serves as a highly efficient promoter to regulate the semihydrogenation performance of Pd active centers. Through the synergistic combination of site isolation and electronic effects, PdS-based catalysts integrate high activity, high selectivity, and broad substrate applicability, representing a core research direction for replacing traditional toxic Lindlar catalysts and enabling a green and efficient alkyne semihydrogenation. In the future, achieving scalable phase-controlled synthesis and precise regulation of S content will be critical to advancing the industrial deployment of these catalytic systems, as this underpins the batch-to-batch performance consistency and long-term operational stability required for practical production.

### 3.5 Multi-Nonmetal-Doped Pd-Based Nanocatalysts for Alkyne Semihydrogenation

As mentioned above, it is well established that introducing nonmetallic elements into Pd-based nanocatalysts can significantly enhance catalytic performance. Recently, some studies have further indicated that multi-nonmetal doping achieves even greater improvements than single nonmetal doping, owing to synergistic effects between different heteroatoms.^136,137^ In the context of alkyne semihydrogenation, such multi-nonmetal doping has shown particular promise. Notably, Wan and coworkers developed B, C codoped Pd anchored on ordered mesoporous carbon, which successfully broke the intrinsic linear scaling relationship (LSR) between alkyne and alkene adsorption energies (Figure 8a–d).^28^ Conventionally, this LSR originates from fixed linear correlations between the adsorption strengths of alkynes and corresponding alkenes, forcing a trade-off between catalytic TOF and alkene selectivity and making it difficult to optimize both simultaneously.^1,138^ In Wan’s work, interstitial doping induced effective charge transfer from B and C to the Pd lattice, increasing the d-electron gain of Pd. This fundamentally altered substrate adsorption configurations and broke the restrictive LSR, thereby enhancing both TOF and selectivity. In the model hydrogenation of 2-methyl-3-butyn-2-ol (MBY) to 2-methyl-3-buten-2-ol (MBE), the B, C-*co*-modified Pd catalysts achieved a 17-fold higher TOF than the Lindlar catalyst while maintaining 95% MBE selectivity at full conversion. The authors attributed this to the fact that the activation entropy for selective conversion of MBY to MBE increases with higher d-electron occupancy of Pd, and the raised d-charge strengthens the interaction between Pd and the C=C bond of MBY, leading to a linear increase in the TOF for MBY conversion (Figure 8e). In contrast, the adsorption of the C=C bond becomes weaker as the d-charge increases, leading to drastically suppressed TOF for MBE hydrogenation. This result is also consistent with DFT calculations (Figure 8f), showing that MBE desorption is energetically more favorable than its further hydrogenation over Pd–B(111), thereby improving selectivity.

**Figure 8:**
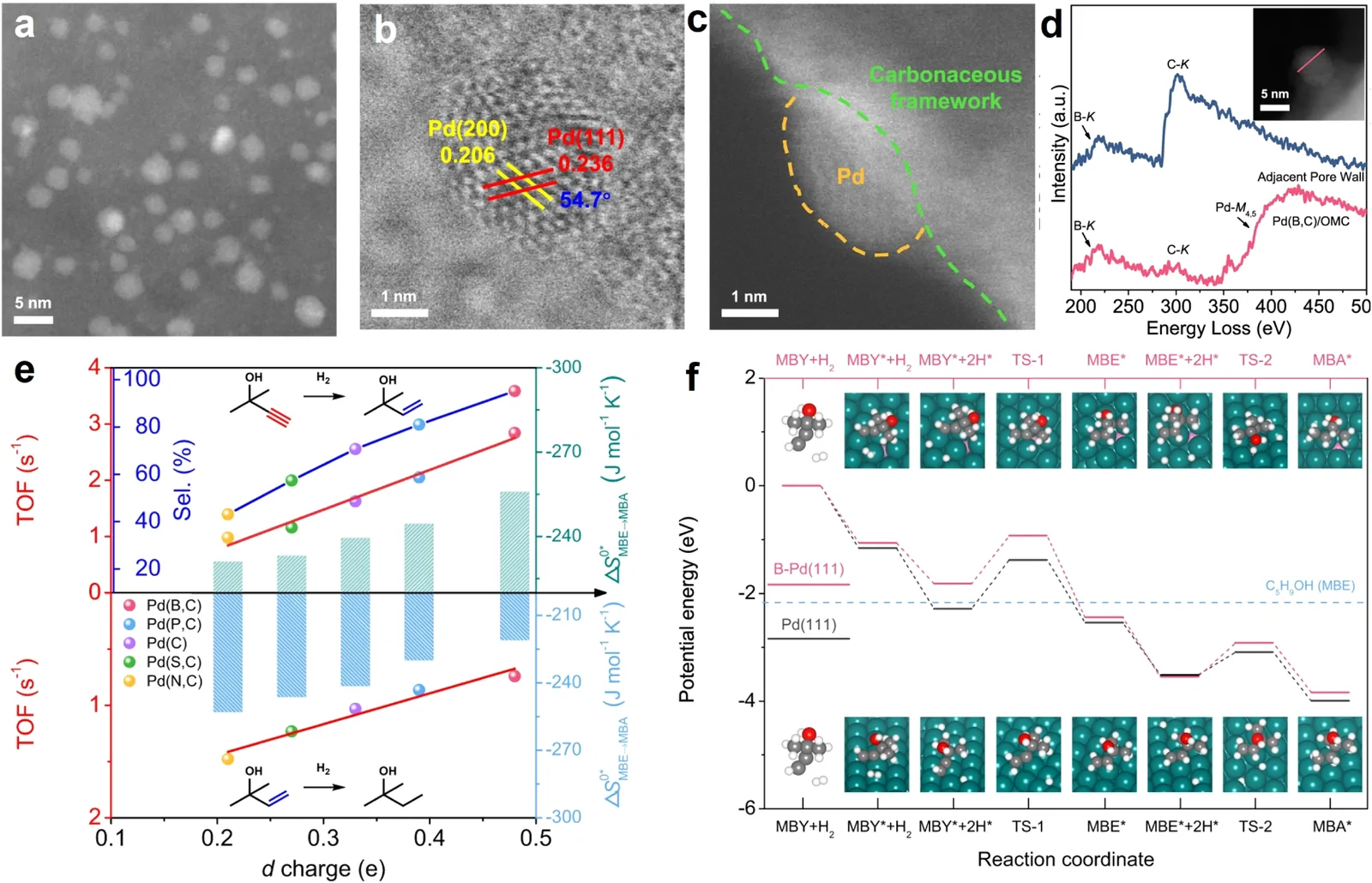
(a–c) Microstructure characterizations of the representative interstitial B, C codoped Pd catalysts, (d) EELS spectra of B, C codoped Pd catalysts. (e) Relationship between the d-charge of Pd sites and TOF (red line)/MBE selectivity (blue line) of the Pd interstitial nanocatalysts, and the relationship between the d-charge of Pd sites and the entropies (ΔS^0*^) for the semihydrogenation of MBY to MBE (light cyan column) or MBE to MBA (light blue column). (f) Reaction pathway for the semihydrogenation of MBY on the pure Pd(111) and B–Pd(111) surfaces. (a–f) Reprinted or adapted with permission under a Creative Commons CC BY license from ref^28^. Copyright 2022 Springer Nature.

Despite the promising synergistic effects demonstrated in reported codoping systems such as Pd–B–C, several fundamental issues remain to be clarified, and research on multi-nonmetal-doped Pd-based catalysts for alkyne semihydrogenation is still in its infancy. First, decoupling the individual catalytic contribution of each dopant remains a common challenge in the field of heterogeneous catalysis. To address this, researchers may benchmark against mono-doped reference samples, employ operando spectroscopy to track the structural evolution of each component under reaction conditions in real time, and combine DFT calculations to differentiate the electronic and geometric effects induced by different heteroatoms.^41^ In addition, it remains unclear whether combinations of multiple nonmetal dopants can definitely produce a reliable synergistic enhancement effect. For hybrid systems containing both small-sized interstitial dopants and large-sized intermetallic compound-forming elements, the spatial proximity between the two types of dopants is regarded as a critical regulatory factor. Closely adjacent dopant configurations facilitate the synergistic modulation of electronic and geometric effects, whereas phase-separated structures may exhibit catalytic performance merely approaching that of a physical mixture.^139^ In terms of dopant quantity, binary nonmetal codoped catalysts have been relatively well reported in this field. Although high-entropy Pd-based materials represent an emerging research direction, whether increasing the number of doping elements necessarily enhances catalytic performance remains to be verified, and a controllable phase structure remains the core prerequisite for performance optimization.^140^ Ternary and higher-order doped systems have rarely been reported for alkyne semihydrogenation and still need further exploration. Future efforts should focus on developing targeted synthesis strategies, combining *in situ* characterization with theoretical calculations to unravel the dynamic roles of multiple dopants, and extending these catalysts to a broader range of alkyne substrates. Addressing these challenges will pave the way for moving beyond isolated model systems to establish general design principles, thereby unlocking the full potential of multi-nonmetal-doped Pd catalysts for efficient and selective alkyne semihydrogenation.

### 3.6 Comparative Summary of Nonmetal-Doped Pd-Based Nanocatalysts for Alkyne Semihydrogenation

Having reviewed the recent progress on nonmetal-doped Pd-based catalysts modified by B, C, P, S, and multi-nonmetal dopants for thermocatalytic alkyne semihydrogenation, we now turn to a comparative summary of these diverse systems to rationalize the distinct differences in their catalytic properties. Since catalytic behavior is dictated by material structure, we first compare available synthetic protocols for producing well-defined nonmetal-doped Pd catalysts, alongside key characterization requirements for validating target doping configurations. Existing strategies encompass gas-phase thermal treatment, high-temperature pyrolysis, and wet-chemical reduction (solvothermal/hydrothermal), with the latter being the most versatile owing to mild conditions and flexible precursor selection. Specifically, boron doping is typically realized via borane-based reductants (*e.g.*, NaBH_4_, DMAB, or BH_3_-THF), where DMAB affords high boron content and induces phase transformation of Pd nanocrystals with well-preserved morphology, despite its high cost and safety concerns.^90–95^ Carbon doping is accessible via decomposition of carbonaceous gases (*e.g.*, C_2_H_4_, C_2_H_2_, or CO) or hydrothermal carbonization of organic precursors (such as glucose); the latter liquid-phase route enables tunable lattice carbon incorporation while retaining the original particle morphology.^87,97–112^ Pd nitride synthesis remains comparatively challenging, with traditional routes requiring extreme high pressure N_2_ or reactive sputtering.^141–143^ Although recent hydrothermal approaches using urea offer milder alternatives, Pd–N systems are still understudied.^144^ For phosphorus-doped Pd, high-temperature decomposition of organophosphorus precursors generates Pd–P intermetallic phases with the risk of organic residue contamination, while inorganic hypophosphite-based phosphorization proceeds under low-temperature conditions, yet precise morphology and crystal-phase control remain difficult.^125–129^ Sulfur-modified Pd materials are obtained from either thermal decomposition of intrinsic S-containing Pd precursors or diverse extrinsic sulfur sources (H_2_S, Na_2_S, thiols, S powder).^122–124,130–135^ Due to the large atomic radius of sulfur, sulfidation predominantly produces Pd–S intermetallic phases rather than pure interstitial doping. The above synthetic comparison highlights that small-sized B, C, and N favor interstitial lattice incorporation under proper conditions, whereas larger P and S heteroatoms thermodynamically tend to form intermetallic compounds, which further underpins the necessity of the complementary characterization described in the above issue.

Obtaining well-defined samples demands a complementary multi-technique characterization workflow to distinguish interstitial incorporation, substitutional modification, and intermetallic second phases. For phase identification and dynamic phase-change monitoring, XRD offers preliminary judgment from lattice-parameter shifts or new diffraction peaks, while operando XRD is recommended to capture structural evolution under realistic reaction conditions. Atomic-scale visualization of interstitial heteroatoms can be realized by aberration-corrected high-angle annular dark-field scanning transmission electron microscopy (HAADF-STEM) combined with electron-energy-loss spectroscopy (EELS) for local elemental mapping. The neutron powder diffraction (NPD) is uniquely effective for locating small interstitial atoms (B, C, N) and their lattice-occupation sites. Electronic structure modification is probed by XANES/EXAFS (bulk averaged bonding and valence information) complemented by surface-sensitive XPS. CO DRIFTS is employed as a molecular probe to interrogate Pd surface ensembles and surface site configurations. Temperature-programmed techniques including H_2_ TPR and H_2_ TPD are essential to evaluate Pd reducibility and hydrogen storage capacity, respectively. Beyond routine laboratory instruments, emerging nonconventional techniques including atom probe tomography (APT), 4D STEM and near ambient pressure XPS (NAP XPS) are highlighted for achieving three-dimensional quantitative dopant mapping, lattice strain analysis and near reaction condition surface characterization. It is worth noting that robust structure-performance conclusions must rely on consistent results from multiple independent characterization tools. Overall, given the distinct properties of various interstitial dopants, we summarize their feasible synthetic routes, essential characterization requirements, dominant structural features, as well as intrinsic advantages and practical limitations for each nonmetallic element in Table 1. This table further offers practical guidance to help readers select appropriate dopants based on their target catalytic performance.

**Table 1:** Comparison of Pd-Based Catalysts Doped with Five Representative Nonmetal Elements for Alkyne Semihydrogenation

**Nonmetal dopant**	**Nonmetallic source**	**Synthetic routes**	**Location within Pd lattice**	**Required characterization evidence**	**Advantages**	**Disadvantages**
Boron (B)	DMAB; NaBH_4_; BH_3_-THF; boric acid	wet chemical reduction; electrochemical deposition	interstitial sites	XRD; XPS; EXAFS (Pd–B bond); STEM-EELS	flexible structure tuning; combined electronic and geometric modulation	costly and hazardous; poor morphology control
Carbon (C)	C_2_H_4_; C_2_H_2_; CO; carbon fiber; glucose	gas-phase thermal treatment; hydrothermal carbonization	interstitial sites	XRD; EXAFS; STEM-EELS; distinguish surface carbon from interstitial carbon	strong Pd–C bonding; suppress *β*-PdH hydride formation	prone to form surface carbon overlayers; risk of undesired phase separation
Nitrogen (N)	N_2_; urea	gas-phase thermal treatment; hydrothermal approach	interstitial or substitutional sites	XRD; EXAFS; STEM-EELS; XPS	strong Pd–N bonding; electronic and geometric lattice modification	narrow synthetic window; prone to phase separation
Phosphorus (P)	PH_3_; NaH_2_PO_2_; TOP; red P; phytic acid	gas-phase thermal treatment; liquid-phase synthesis	substitutional sites; Pd–P intermetallic phases	XRD (to identify intermetallic/amorphous phase) EXAFS; STEM-EELS	intermetallic-based ensemble dilution effect; induce lattice distortion	risk of residual organic contamination; poor control over morphology
Sulfur (S)	H_2_S; Na_2_S; S powder; methylene blue	gas-phase thermal treatment; liquid-phase synthesis	substitutional sites; Pd–S intermetallic phases	XRD (intermetallic phase); EXAFS; STEM-EELS	strong Pd–S interaction; effective ensemble-dilution effect	may poison active sites; hard to precisely fine-tune S dopant

Broadly speaking, small-sized B and C tend to occupy interstitial sites within the Pd lattice. By contrast, larger-radius P and S prefer substitutional positions and readily form intermetallic phases. N-doped Pd systems suffer from narrow synthetic windows and remain largely underexplored for thermocatalytic alkyne semihydrogenation. With well-defined structural information accessible from experimental characterizations, DFT calculations can further offer mechanistic theoretical insights into electronic and geometric modulation induced by subsurface heteroatoms. As for the theoretical insights, Wu *et al.* conducted a comprehensive DFT and microkinetic study on six subsurface heteroatoms (H, B, C, N, P, S).^62^ Their findings reveal that the electronegativity of dopants determines the charge transfer between nonmetal and Pd and further modulates the d-band structure (Figure 9a), while all nonmetallic dopants can suppress the formation of *β*-PdH *via* geometric confinement. The theoretically predicted performance trend shows that electronic and geometric effects play a dominant role together in Pd–B_0.5_ and Pd–C_0.5_ catalysts, while the geometric effect is the key contributor in Pd–N, Pd–P, and Pd–S catalysts. For the C_2_H_4_ selectivity, the order follows Pd–C_0.5_ > Pd–B_0.5_ > Pd–S > Pd–N > Pd–H_1.0_ > Pd–P > Pd (Figure 9b). These theoretical predictions not only corroborate the mechanistic understanding of electronic and geometric modulation but also provide a valuable descriptor for the rational design of high-performance nonmetal-doped Pd catalysts.

**Figure 9:**
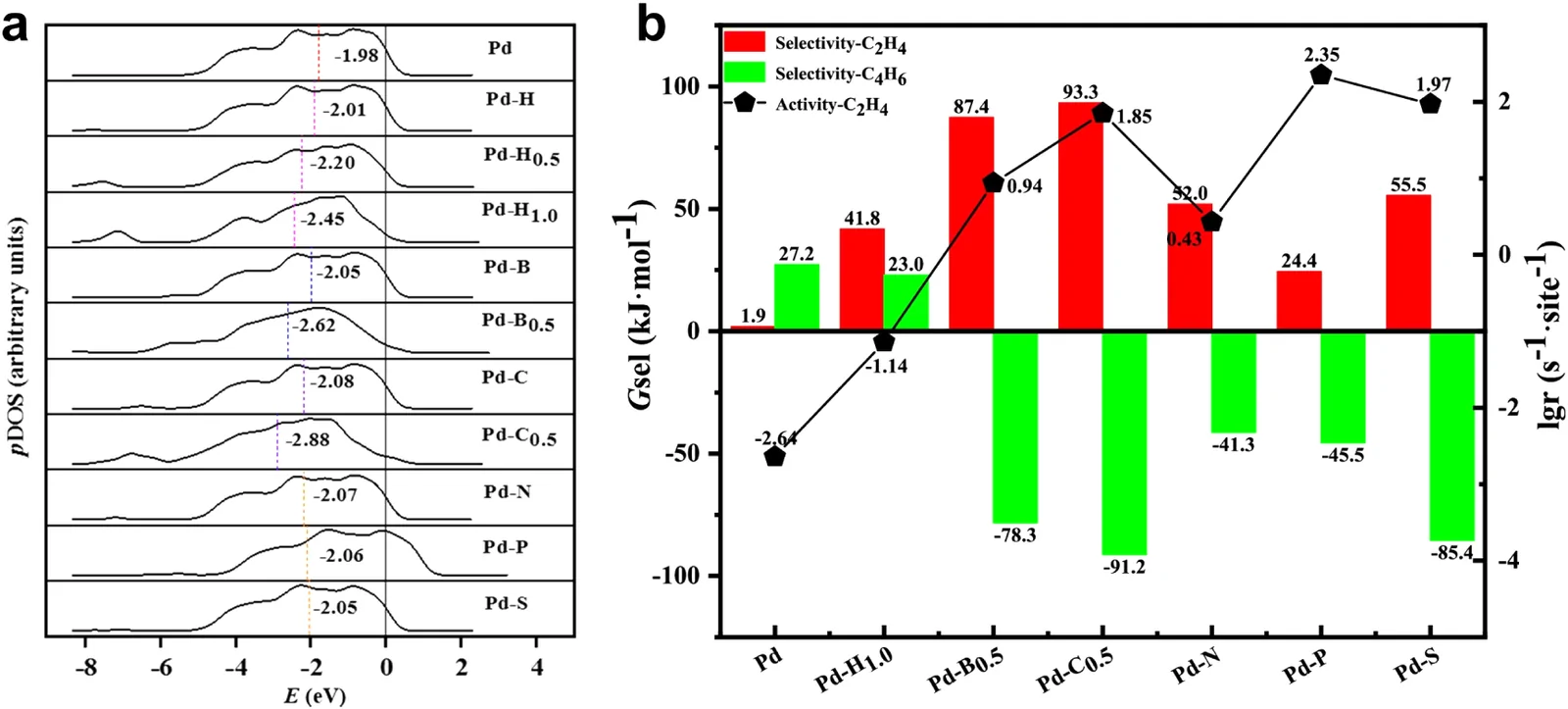
(a) Projected density of states (pDOS) of d-orbitals for surface Pd atoms over subsurface nonmetal-doped Pd catalysts. (b) Theoretical predictions of C_2_H_4_ and C_4_H_6_ selectivity, along with C_2_H_4_ activity for subsurface nonmetal-doped Pd catalysts. Reproduced with permission from ref^62^. Copyright 2022 American Chemical Society.

To further distill general structure-performance relationships from these diverse systems, it is instructive to directly compare their catalytic performance under analogous reaction conditions. Table 2 summarizes the key reaction parameters of the representative catalysts discussed above, enabling a straightforward comparison of their activity and selectivity. Moreover, to facilitate a more intuitive comparison of the data presented in Table 2, we present a selectivity-versus-conversion plot that positions the five nonmetal-doped catalysts relative to industrial benchmarks and state-of-the-art Pd alloyed systems in Figure 10. For industrial acetylene hydrogenation, the residual acetylene concentration must be reduced to below 5 ppm (or even lower) to meet polymer-grade ethylene specifications, corresponding to an acetylene conversion exceeding 99.5%.^40^ Ethylene selectivity must be maintained at the highest possible level under such demanding conversion requirements. Compared with state-of-the-art Pd alloyed catalysts (*e.g.*, PdAg, PdZn, and PdGa), nonmetal-doped systems offer competitive conversion and selectivity with the additional advantages of lower cost and simpler synthesis. Given the rapid progress in this field, further enhancements in catalytic performance are anticipated through continued optimization of synthetic strategies and exploration of a broader range of nonmetal-doped Pd systems.

**Table 2:** Representative Nonmetal-Doped Pd-Based Catalysts for Thermocatalytic Alkyne Semihydrogenation

**Catalyst**	**Reaction phase**	**Model substrate**	**Reaction condition**	**Selectivity to alkene (%)**	**Conversion of alkyne (%)**	**TOF (h** ^**–1**^)	**Ref**
Pd-^int^C	liquid-phase	3-hexyn-1-ol/2-butyn-1,4-diol	30–50 °C, 3–10 bar	100%	91.9%/97.2%	NA	^88^/^100^
PdC_0.18_ nanocubes	liquid-phase	4-ethynyl-1,1′-biphenyl	30 °C, 1 bar	100%	>99%	7896	^24^
PdC/Al_2_O_3_	gas-phase	acetylene	240 °C, 0.1 MPa,	100%	92.8%	NA	^90^
Pd_3_ZnC_0.03_/Al_2_O_3_	gas-phase	acetylene	60 °C, 0.5 MPa,	94%	91%	1872	^87^
PdC_*x*_/Al_2_O_3_	gas-phase	acetylene	120 °C, 0.1 MPa,	98%	92%	NA	^101^
Pd-^int^B/C	gas-phase	acetylene	75 °C, 1 bar	>90%	>80%	NA	^23^
Pd-^int^B	gas-phase	acetylene	75 °C, 1 bar	>95%	>90%	NA	^88^
Pd_2_B/Al_2_O_3_	liquid-phase	3-hexyne-1-ol	30 °C, 0.1 MPa	99.8%	95.9%	NA	^80^
Pd–P nanoparticles	liquid-phase	diphenylacetylene	30 °C, 1 bar	100%	95–98%	180	^107^
Pd_3_P/TiO_2_	gas-phase	acetylene	75–225 °C, 1 bar	100%	78%	850	^26^
PdP_2_/TiO_2_	gas-phase	acetylene	75–225 °C, 1 bar	100%	85%	1200	^26^
Pd_3_P_0.95_/SiO_2_	liquid-phase	phenylacetylene	RT, 1 bar	>99%	99%	NA	^119^
meso-PdP nanobundles	liquid-phase	1-phenyl-1-propyne	60 °C, ambient	100%	97.9%	23.38	^112^
Pd_3_S/C_3_N_4_	liquid-phase	2-methyl-3-butyn-2-ol	303 K, 1 bar	100%	100%	1200	^27^
Pd_4_S/CNF	gas-phase	1-butyne/2-butyne	473 K, 1 bar	100%	92–93%	1800	^124^
h-meso PdS	liquid-phase	4-nitrophenylacetylene	40 °C, ammonia borane as H-donor	100%	94%	NA	^125^
bulk Pd_4_S	gas-phase	acetylene	250 °C, 1 bar	100%	90.9%	850	^122^
Pd_30_Cu_10_S_9_	liquid-phase	diphenylacetylene	RT, silane as H-donor	>99%	97%	320	^126^
B, C-co doped Pd	liquid-phase	2-methyl-3-butyn-2-ol	25 °C, 1 bar	95%	∼100%	10224	^28^

**Figure 10:**
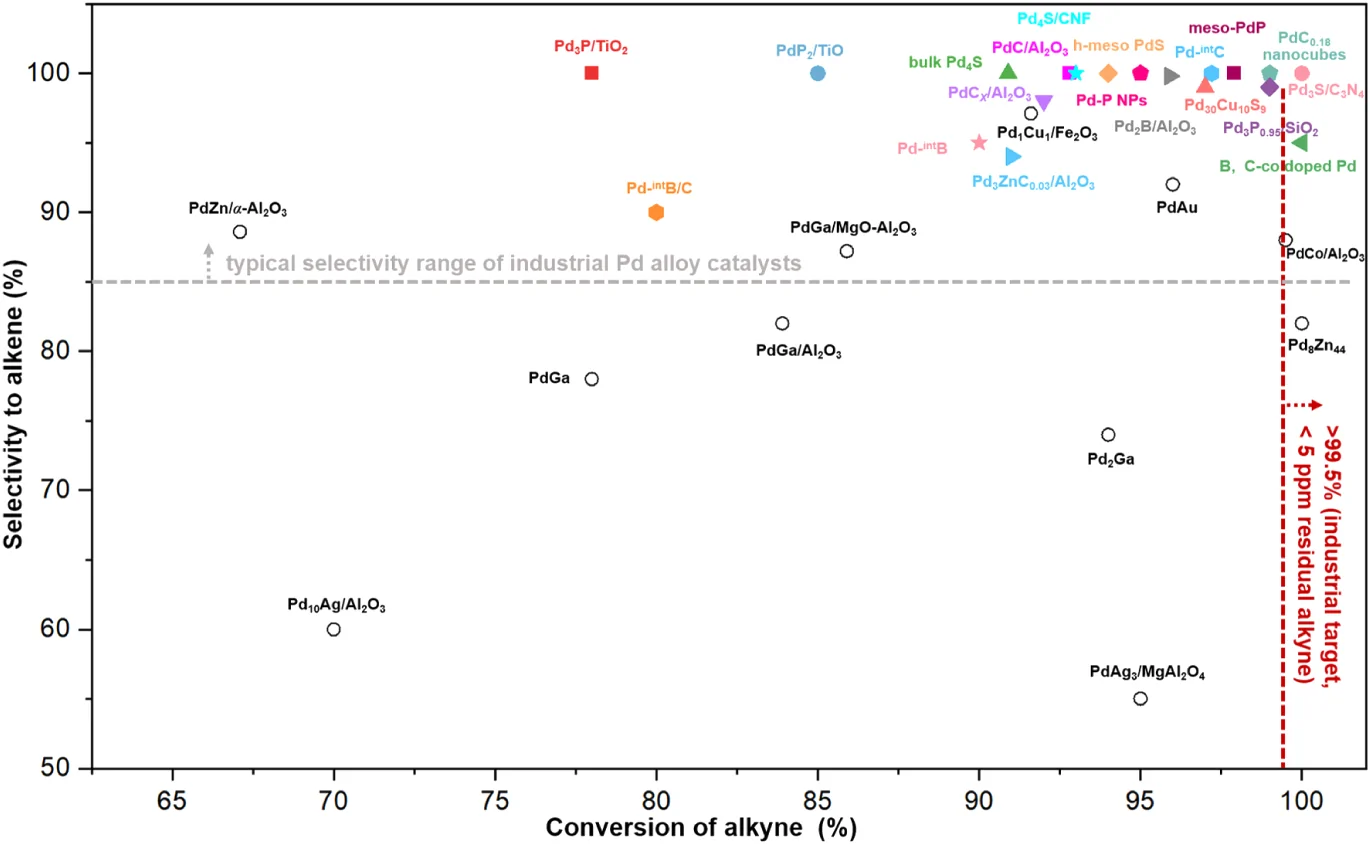
Alkene selectivity versus alkyne conversion for representative nonmetal-doped Pd catalysts (solid symbols) and state-of-the-art Pd alloy catalysts (open circles) according to Table 2. The vertical dashed line denotes the industrial target of >99.5% alkyne conversion (corresponding to <5 ppm residual acetylene for polymer-grade alkene production). The horizontal dashed line indicates the typical selectivity range of industrial Pd-alloy catalysts.

## 4 Interstitial Nonmetal Doping of Pd Nanocatalysts for Electrocatalytic Alkyne Semihydrogenation

### 4.1 Mechanistic Insights into Electrocatalytic Hydrogenation

Electrocatalytic alkyne hydrogenation employs H* generated via electric-driven H_2_O splitting as the hydrogen source. In this system, two distinct reaction pathways govern the electrocatalytic hydrogenation of alkynes (Figure 11a).^145^ The first is the proton-coupled electron transfer (PCET) mechanism, in which alkyne reactants are initially adsorbed on the catalyst surface. Subsequently, surface-bound species undergo protonation by electrolyte-derived H^+^ or H_2_O, accompanied by synchronous electron transfer, and the final hydrogenated products eventually desorb from catalytic sites. Recent kinetic isotope effect measurements and radical intermediate identification have further verified that the hydrogen atom transfer (HAT) mechanism dominates the semihydrogenation of alkynes.^146–148^ This mechanism proceeds through four core steps (Figure 11c): (1) selective chemisorption of alkyne molecules on the catalyst surface; (2) *in situ* generation of H* via the Volmer reaction (H_2_O + M + e^–^ → M–H* + OH^–^); (3) preferential hydrogenation of the adsorbed C≡C triple bond by electrophilic H* to form alkene intermediates; (4) desorption of target alkene products and their diffusion into the bulk electrolyte.^149^ The kinetics of heterogeneous electrocatalytic reactions are fundamentally determined by reactant adsorption at the catalyst and electrolyte interface, which acts as the essential precursor for subsequent electron transfer and bond rearrangement. Among diverse catalytic materials, Pd-based catalysts exhibit prominent intrinsic activity toward C≡C bond activation.^150,151^ For electrocatalytic systems, Pd also possesses excellent water dissociation capability, as reflected by the favorable Gibbs free energy of hydrogen adsorption (ΔG_H*_) profiles in Figure 11b, which enables efficient generation of reactive hydrogen intermediates (H*).^152^ Owing to its specific electronic structure, Pd facilitates facile dissociation of water molecules into surface-adsorbed H* at a relatively low potential (*e.g.*, 0.4 V vs. RHE).^150^ The *in situ* generated H* immediately reacts with adjacent adsorbed alkyne species, thereby driving the semi-hydrogenation process. Overall, the synergistic combination of efficient alkyne adsorption/activation and favorable water dissociation kinetics renders Pd a state-of-the-art benchmark catalyst for electrocatalytic selective alkyne hydrogenation.

**Figure 11:**
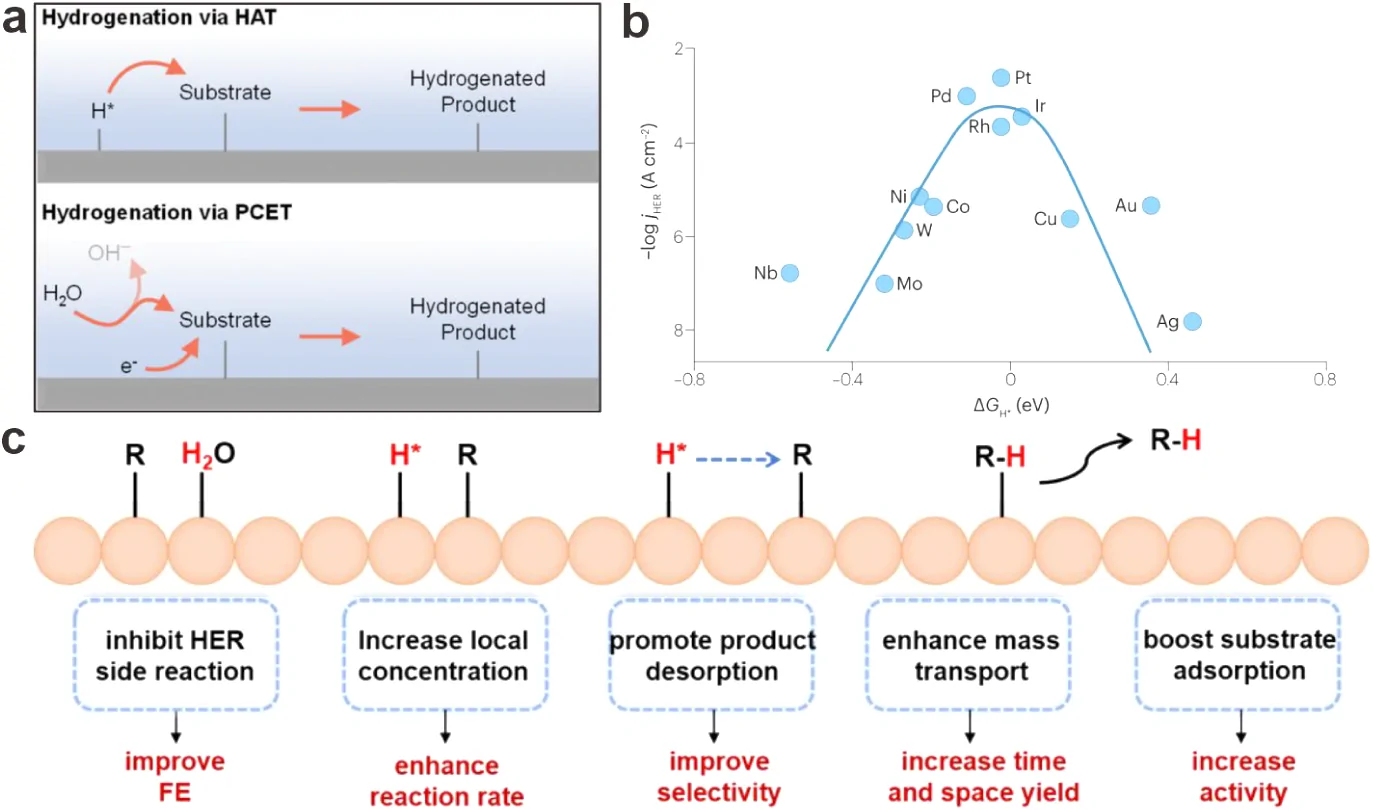
(a) Schematic diagram of alkyne semihydrogenation reaction mechanisms. Reproduced with permission from ref^146^. Copyright 2024 Royal Society of Chemistry. (b) Exchange current densities (−log­(j_HER_)) *vs.* ΔG_H*_ for HER over different electrodes. (a, b) Reproduced with permission from ref^153^. Copyright 2024 Springer Nature. (c) Typical reaction steps and consideration factors for electrocatalytic alkyne semihydrogenation with H_2_O.

A major bottleneck restricting aqueous-phase electrocatalytic alkyne semihydrogenation is the intrinsic interfacial competition between target alkyne hydrogenation and parasitic hydrogen evolution reaction (HER). The *in situ* generated adsorbed H* intermediates can be consumed to generate H_2_ through either the Tafel pathway (H* self-recombination) or the Heyrovsky pathway (H* + H_2_O + e^–^ → H_2_ + OH^–^).^77,149^ Such competitive side reactions drastically consume H* species that are supposed to drive alkyne semihydrogenation, thereby lowering the reaction efficiency of the desired alkene product. Although Pd-based catalysts exhibit excellent capability for H_2_O activation, this superiority simultaneously triggers severe HER competition, particularly under high overpotential conditions. Furthermore, Pd possesses strong binding affinity toward unsaturated chemical bonds, which gives rise to two unfavorable phenomena: competitive surface adsorption between alkyne substrates and H* species, as well as the difficult desorption of hydrogenation intermediates from catalyst surfaces. These drawbacks synergistically suppress substrate conversion efficiency and alkene product selectivity.

### 4.2 Electronic Effect Modified by Nonmetal Doping for Electrocatalytic Alkyne Semihydrogenation

Existing studies have demonstrated that the electrocatalytic semihydrogenation performance of alkynes is predominantly governed by the surface adsorption intensity of various reaction species and the adsorption geometry of reactants and intermediates. Both pivotal catalytic descriptors can be effectively regulated by tuning the electronic structure and atomic coordination environment of active sites. Benefiting from their distinct electronegativity and small atomic radius, nonmetal atoms preferentially enter the Pd lattice, which induces lattice strain and modulates the electronic structure of local active sites, resulting in accelerated reaction kinetics and improved semihydrogenation selectivity of the catalytic system. In recent years, abundant experimental results and theoretical calculations have consistently validated that nonmetallic heteroatom doping serves as a powerful strategy to significantly boost the catalytic performance of Pd-based catalysts toward alkyne semihydrogenation.^28,112,153–157^ For example, Zhang *et al.* first pioneered the application of P-doped Pd catalysts for electrocatalytic semihydrogenation and deuteration of alkynes.^153^ DFT simulations reveal that P doping in Pd–P nanowires (Pd–P NWs) simultaneously strengthens alkyne adsorption and promotes water-splitting kinetics for *in-situ* H* generation (Figure 12a,b). Benefiting from such dual optimization, the Pd–P NWs catalysts achieve a high product selectivity of 99% and a FE of 78% at low applied potentials, distinctly superior to pure Pd counterparts (Figure 12c). In another study, Li’s group introduced low-electronegativity and small-sized B atoms into the bulk lattice of Pd catalysts (Figure 12d).^29^ Precise modulation of the B doping level optimizes interfacial charge transfer kinetics and induces significant surface electron density redistribution over Pd active sites. The reconstructed electronic structure preferentially accelerates the targeted semihydrogenation of MBY to MBE, while suppressing the undesired overhydrogenation pathway toward alkanol products (Figure 12e). At −1.0 V *vs.* RHE, the B-doped Pd catalyst achieves a high MBY conversion of 94.5% and an MBE selectivity of 90.2%, with a calculated TOF of 139.8 h^–1^. This value is 1.28 and 1.93 times higher than those of pure Pd and commercial Pd/C, respectively. Nevertheless, the severe competitive HER at such high overpotentials restricts the FE to only 39% (Figure 12f). Apart from P and B doping, S modulation represents another effective strategy for electronic structure engineering of Pd catalysts. In thermal catalysis, S doping has been proven to optimize HER activity and semihydrogenation selectivity.^30,157^ Furthermore, Zhang’s team advanced this by developing a sulfur-modified, carbon-supported high-curvature Pd nanotip catalyst (Pd@ArS-Pd_4_S NTs), supported by combined experiments and DFT calculations.^154^ The calculations indicate that lattice S^2–^ weakens alkene adsorption, while surface thiolates lower the water activation barrier and ΔG_H*_. This design synergizes the nanotip’s enrichment effect with S-induced adsorption modulation: facile H* generation boosts conversion, and weak alkene binding promotes product desorption. The optimized catalyst achieved >92% alkyne conversion, >90% alkene selectivity, and >67% FE at −1.0 to −1.3 V *vs.* Hg/HgO.

**Figure 12:**
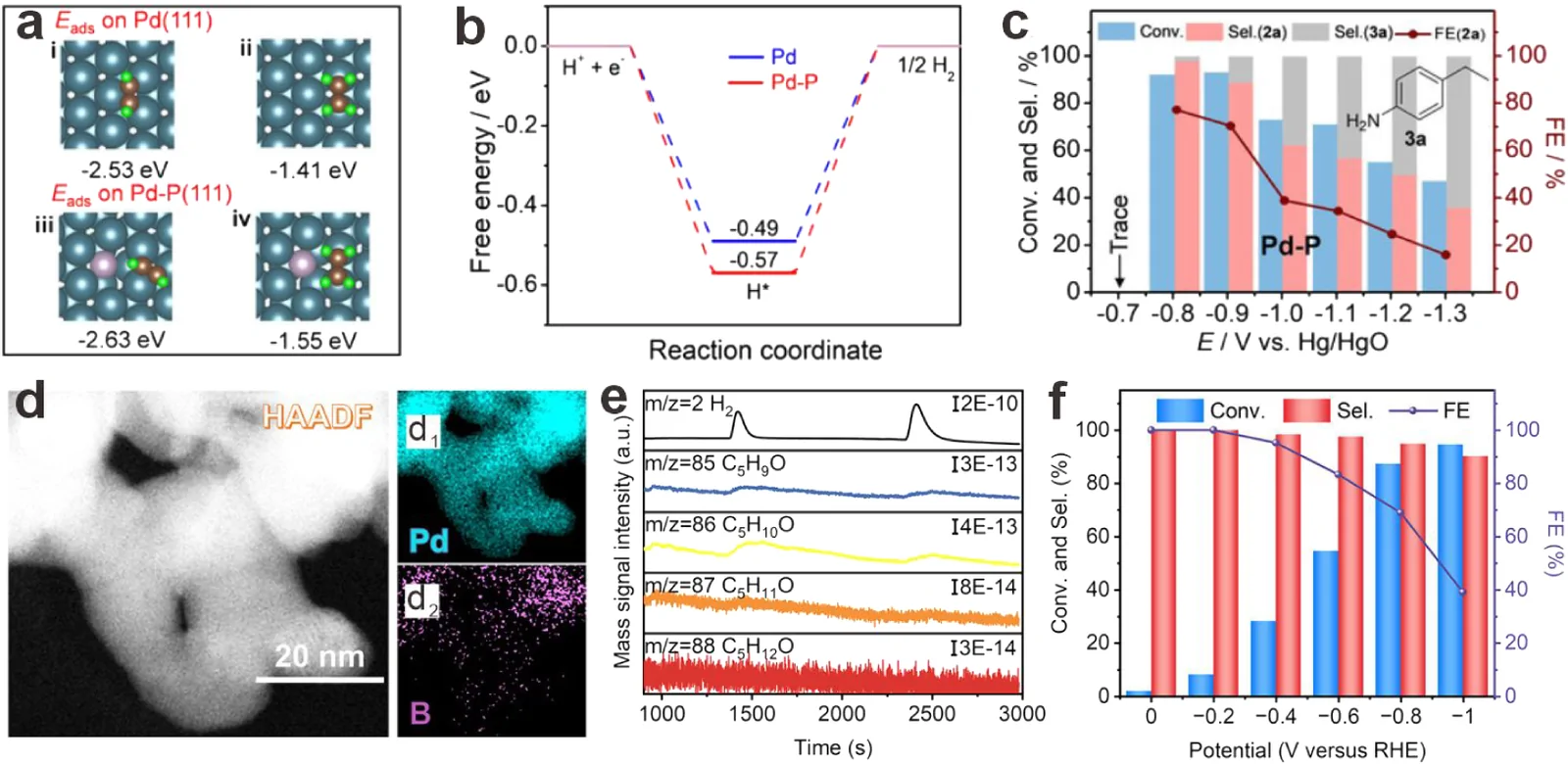
(a) DFT calculations of adsorption energy of acetylene and ethylene over Pd–P and pure Pd catalysts. (b) DFT calculations of free energy diagram for hydrogen adsorption on Pd–P and Pd. (c) Alkene selectivities and FEs over Pd–P NNs under different potentials. (a–c) Reproduced with permission from ref^154^. Copyright 2020 Wiley-VCH. (d) EDX elemental mapping profiles of PdB catalyst with Pd (d_1_) and B (d_2_) distribution. (e) Online differential electrochemical mass spectrometry (DEMS) measurements of MBY electroreduction over the PdB catalyst. (f) Electrocatalytic MBY semihydrogenation performance on the PdB catalyst under different potentials. (d–f) Reproduced with permission from ref^29^. Copyright 2023 American Chemical Society.

### 4.3 Geometric Effects Modified by Nonmetal Atoms for Electrocatalytic Alkyne Semihydrogenation

Beyond the above results, substantial studies have verified that nonmetallic doping can precisely tailor the electronic structure of Pd catalysts via unique s-p-d orbital hybridization, offering a reliable strategy to modulate the surface adsorption and desorption behaviors that dominate alkyne hydrogenation.^158,159^ Moreover, large-radius dopants such as P and S tend to either occupy Pd lattice interstitial sites or substitute for lattice Pd atoms, which are highly dependent on the specific synthetic and modification strategies. Given such structural diversity, exploring the synergistic electronic and geometric modulation effects of nonmetallic doping has become a research hotspot for designing high-efficiency Pd-based electrocatalysts toward selective alkyne hydrogenation. For instance, Luo *et al.* developed a lattice sulfur-induced amorphization strategy to directly grow composition-tunable, disordered palladium sulfide nanosheets (PdS_*x*_ NSs) on nickel foam.^31^ In terms of electronic modulation, the incorporation of S into the Pd lattice generates electron-deficient Pd active centers, which weaken the adsorption affinity toward alkene products and thereby improve catalytic selectivity. Previous studies have confirmed that sulfur species can adsorb hydrogen intermediates and promote HER.^160^ Notably, lattice S incorporation triggers the amorphous transformation of Pd nanosheets, which fundamentally alters the local coordination environment of Pd atoms from a geometric perspective. The resulting disordered atomic arrangement substantially increases the number of exposed active sites and enhances the intrinsic catalytic activity. These abundant active centers mainly consist of coordination-unsaturated surface Pd atoms, homogenized tiny Pd ensembles with isotropic electronic properties, and intrinsic structural defects within the metastable disordered matrix.^161–163^ More importantly, lattice sulfur regulation balances the competitive adsorption between alkynes and H* intermediates on the catalyst surface. This effect effectively suppresses the unfavorable H* coupling side reactions, thereby greatly improving alkene FE. The combined electronic and geometric modulation of Pd active sites by lattice sulfur endows the optimized PdS_*x*_ catalyst with excellent electrocatalytic performance, achieving approximately 99% substrate conversion, 97% alkene selectivity, and 94% FE at a low potential of −0.298 V vs. RHE.

Moreover, geometric effects induced by nonmetallic doping were also applied for achieving high chemoselectivity and regioselectivity for alkynes containing highly reducible bonds or strongly coordinating functional groups. Zhang *et al.* developed amorphous PdS_*x*_ nanocapsules (PdS_*x*_ ANCs) with abundant unsaturated, isolated Pd sites via sulfur modification (Figure 13a,b).^85^ The introduced S dopant realizes dual-functional modulation in both electronic and geometric aspects (Figure 13c). The Pd centers substantially weaken the binding strength of alkene products, while the disordered amorphous framework induces a distinctive σ-alkynyl adsorption configuration (Figure 13d). This unique adsorption mode enlarges the spatial distance between the substrate’s auxiliary functional groups and the catalyst surface, effectively avoiding undesired reduction or coordination-induced deactivation. Benefiting from the synergistic electronic and geometric regulation, the PdS_*x*_ ANCs catalyst exhibits >95% conversion of 4-ethynylbenzamide within a potential window of −1.05 V to −1.30 V, together with an excellent alkene selectivity of up to 96% (Figure 13e).

**Figure 13:**
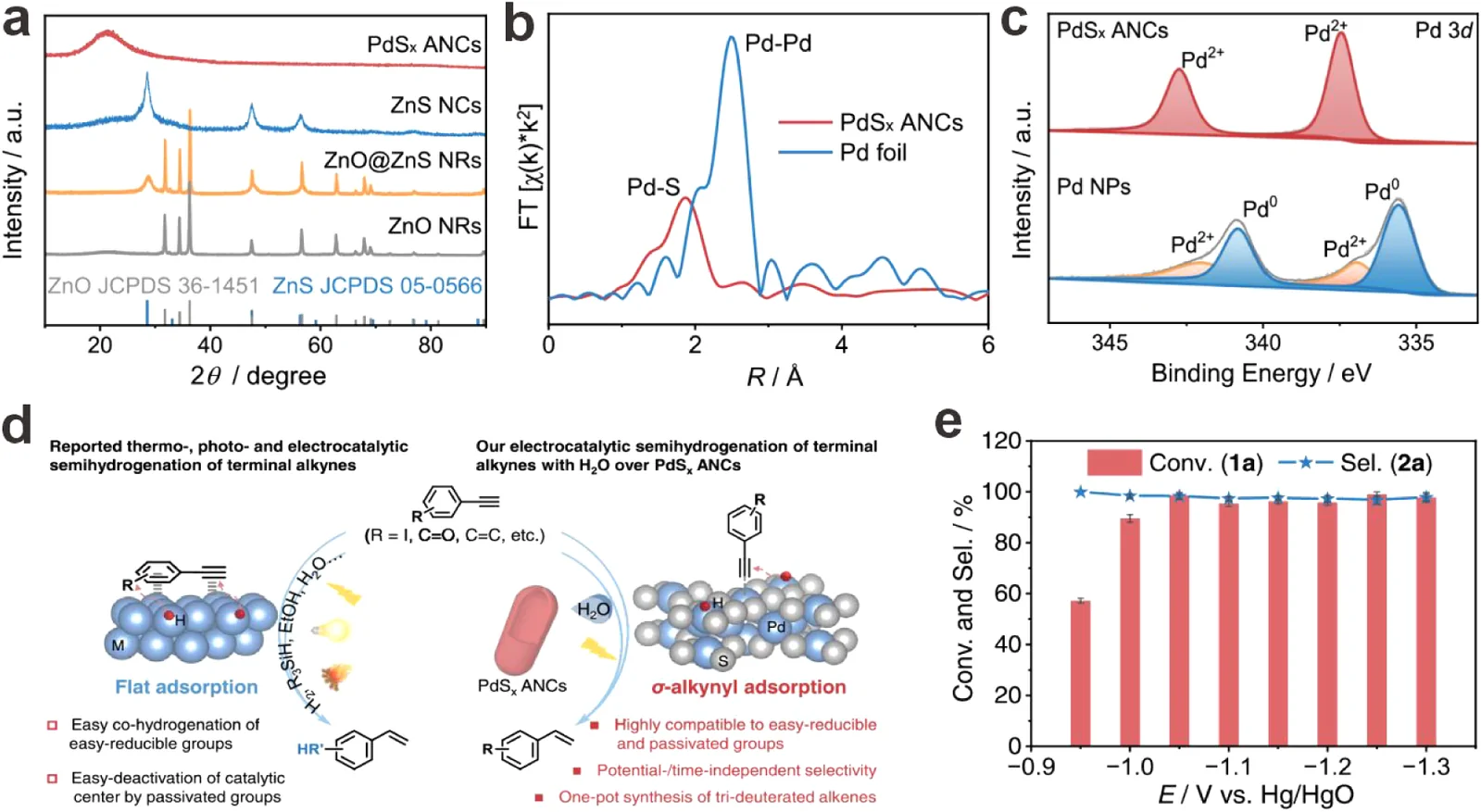
(a) XRD patterns of PdS*_x_* ANCs catalysts. (b) FT-EXAFS spectra of PdS*_x_* ANCs catalysts. (c) XPS of Pd 3d for PdS*_x_* ANCs and Pd NPs. (d) Schematic diagram of adsorption mode in electrocatalytic semihydrogenation of terminal alkynes with readily reducible and passivated functional groups. (e) Electrocatalytic hydrogenation performance of 4-ethynylaniline over PdS*_x_* ANCs within 4 h. (a–e) Reproduced with permission from ref^85^. Copyright 2022 American Chemical Society.

For a Pd-cathode-based electrocatalytic alkyne hydrogenation, the dynamic generation and surface coverage of adsorbed H* intermediates are as vital as the regulated adsorption of substrates and intermediates for catalytic performance optimization.^164^ As a decisive intermediate, surface H* governs the branching of hydrogenation reaction pathways and directly determines the FE of target alkene products. With small atomic size and excellent lattice compatibility with Pd, nonmetallic atoms with small radii have also emerged as efficient modulators for high-performance electrocatalytic alkyne hydrogenation. As described above, these doped nonmetallic atoms preferentially occupy the interstitial voids of the Pd lattice, delivering dual regulatory effects. Structurally, such occupation suppresses the formation of subsurface hydrogen species originating from the intrinsic hydrogen storage behavior of Pd, thus avoiding excessive hydrogenation. Electronically, strong orbital interaction between dopants and neighboring Pd sites remodels the electron distribution of local active centers and optimizes catalytic interfacial properties.

Currently, the dual-functional modulation of nonmetallic doping, which integrates electronic structure engineering and atomic-level steric hindrance regulation, has become a rapidly advancing research frontier in the field of electrocatalytic alkyne semihydrogenation. Zhang’s research group reported a nitrogen-doped Pd nanocube catalyst (Pd_2_N NCs) that comprehensively enhances the catalytic performance of Pd-based electrocatalytic alkyne hydrogenation.^25^ Structural characterization confirms that the doped N atoms selectively occupy the octahedral interstitial sites of the Pd lattice (Figure 14a). Such site-preferential N doping effectively suppresses the generation of PdH_*x*_ subsurface species while fully preserving the surface-active Pd sites for catalytic reactions. Combining experimental results and DFT calculations demonstrates that the improved alkene selectivity stems from the synergistic modulation of electronic structure and lattice interstitial steric hindrance (Figure 14b). The strong orbital hybridization between highly electronegative N atoms and adjacent Pd centers induces remarkable surface electron redistribution, which downshifts the d-band center of Pd active sites, thereby weakening excessive alkene intermediate adsorption and significantly raising the energy barrier for the over-hydrogenation pathway. Furthermore, the optimized electronic structure renders the H* adsorption energy on the Pd_2_N NCs surface higher than the energy barrier for alkyne hydrogenation (Figure 14c). This favorable thermodynamic regulation efficiently restrains the competitive HER and further promotes target alkene selectivity. Consequently, Pd_2_N NCs achieve a high alkyne conversion of 95.7%, alkene selectivity of 97.8%, and FE of 72.4% at −1.1 V *vs.* Hg/HgO. Inspired by this, our results demonstrate that carbon-induced stereoscopic steric repulsion efficiently blocks the penetration of hydrogen atoms into the Pd subsurface and inhibits hydride formation.^140^ Moreover, interstitial carbon doping moderately downshifts the d-band center of Pd, which alleviates the over-adsorption of alkene products (Figure 14d,e). Benefiting from such dual optimization, the optimized PdC_*x*_ NCs exhibit more than twice the alkene selectivity relative to pristine Pd nanocubes within a wide potential window of −0.9 V to −1.4 V (*vs.* Hg/HgO), while completely preserving the high intrinsic catalytic activity of pristine Pd catalysts (Figure 14f).

**Figure 14:**
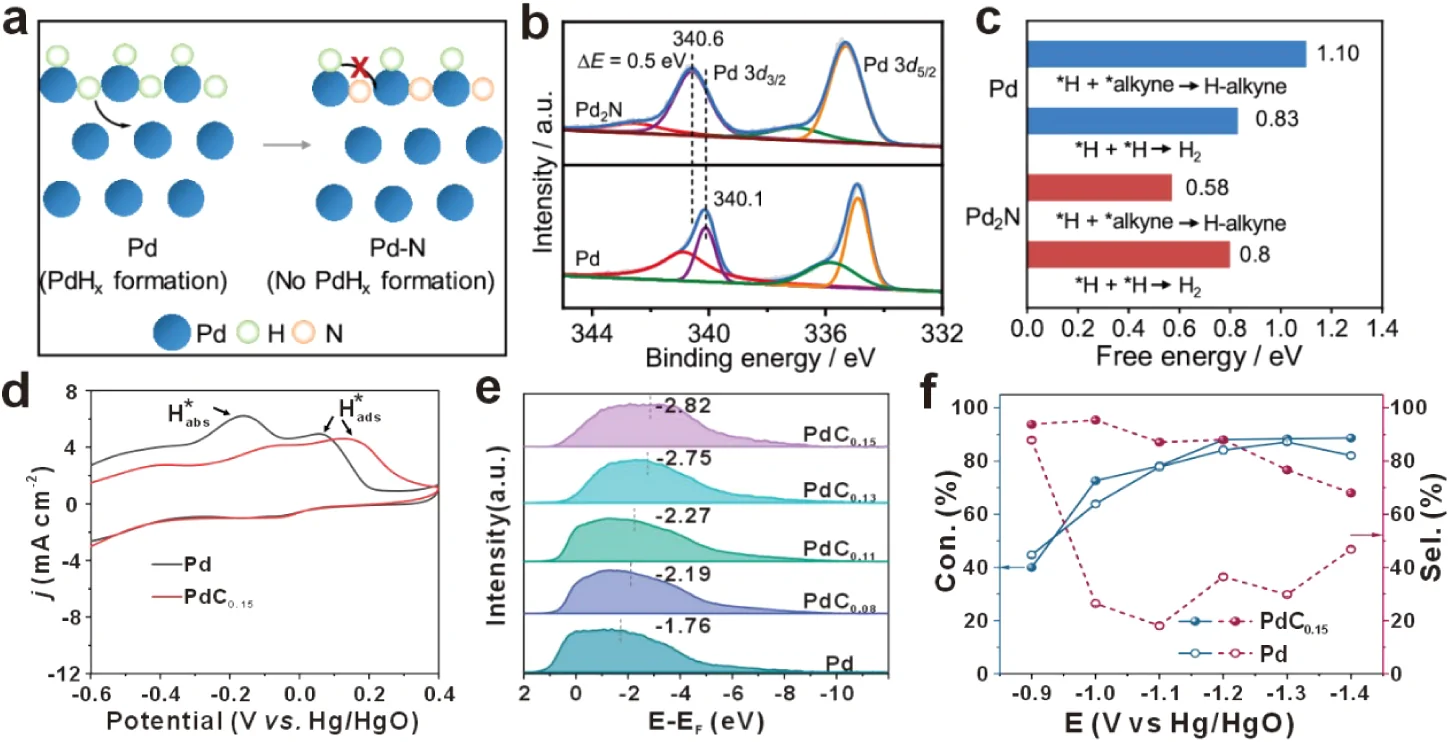
(a) Proposed PdH*_x_* inhibition mechanism for PdN*_x_*. (b) XPS of Pd 3d for Pd and Pd_2_N NCs. (c) Free energy for active hydrogen self-coupling and self-addition to an alkyne molecule. (a-c) Reproduced with permission from ref^25^. Copyright 2023 American Chemical Society. (d) Cyclic voltammetry (CV) profiles of PdC_0.15_ and pure Pd NCs in a 0.5 M Na_2_SO_4_ solution. (e) High-resolution valence band XPS of Pd and PdC*_x_* NCs. (f) Comparative electrochemical semihydrogenation performance of Pd and PdC_0.15_ NCs. (d–f) Reproduced with permission from ref^156^. Copyright 2025 American Chemical Society.

Conventional heteroatom modification of monometallic Pd usually triggers synchronous variation in the adsorption energies of both alkynol substrates and alkenol products. Such inherent limitation makes it challenging to balance catalytic activity and product selectivity in electrocatalytic hydrogenation. Different from the aforementioned doping modulation of pure Pd, taking full advantage of the electronic regulatory effect of carbon doping, our research group further developed a carbon-modified Pd_78_Cu_22_ alloy catalyst to construct dual active centers comprising electron-deficient Pd^δ+^ sites and electron-rich Cu^0^/Cu^δ−^ sites (Figure 15a, b).^32^ The generated Pd centers can synergistically stabilize adsorbed H* and promote the preferential adsorption of alkynol molecules, thereby guaranteeing efficient substrate conversion. Meanwhile, Cu^0^/Cu^δ−^ sites repel C=C bond-containing alkenol products through electrostatic interaction, which facilitates rapid product desorption and achieves high alkenol selectivity. Moreover, CV tests confirm that the interstitial carbon atoms in the alloy lattice effectively suppress the formation of unfavorable PdH_*x*_ species (Figure 15c).

**Figure 15:**
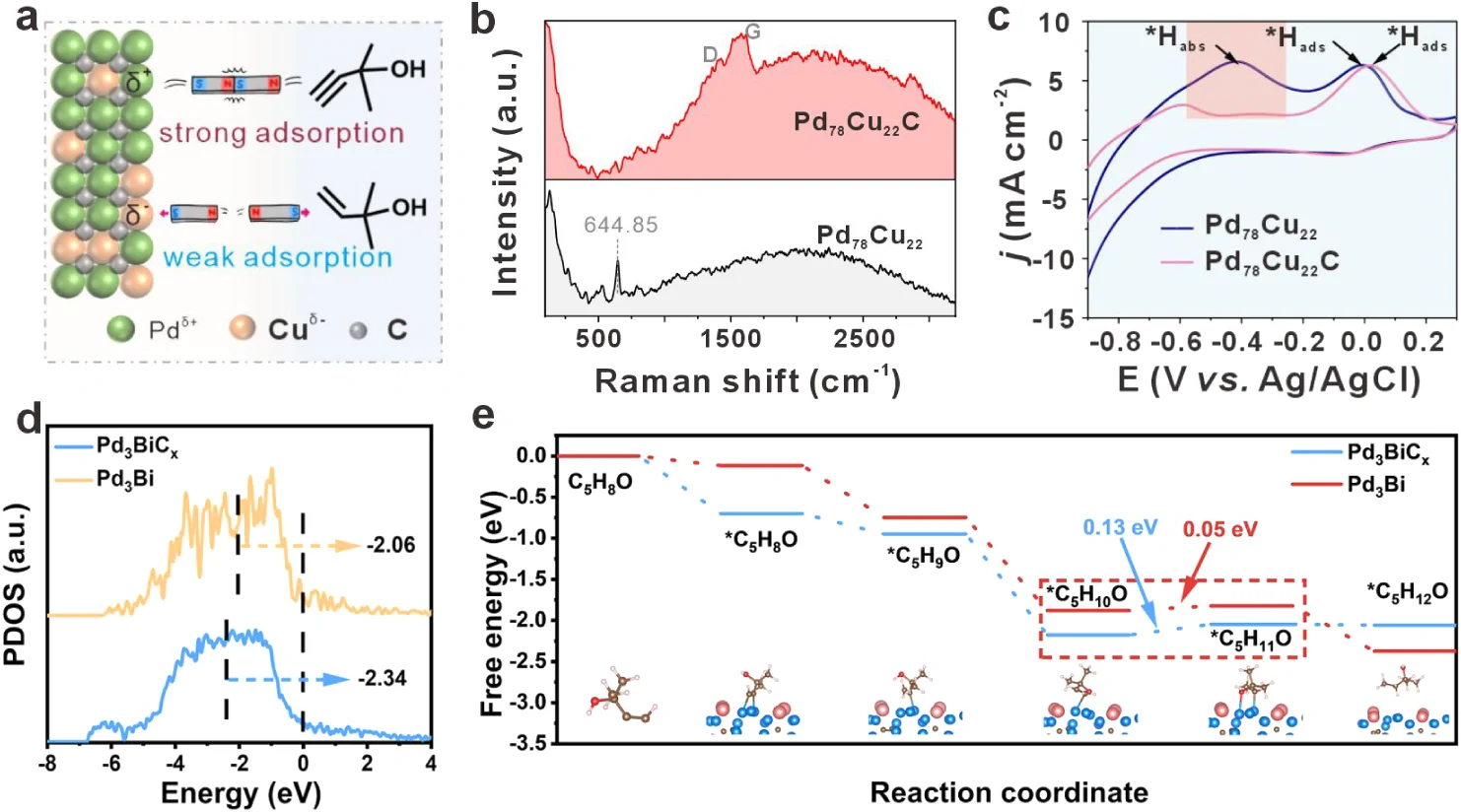
(a) A schematic illustration of the differential adsorption mechanism for MBY and MBE. (b) Raman spectra of Pd_78_Cu_22_C NFs and Pd_78_Cu_22_ NFs. (c) CV profiles of Pd_78_Cu_22_C NFs and Pd_78_Cu_22_ NFs in a 0.5 M Na_2_SO_4_ solution. (a–c) Reproduced with permission from ref^32^. Copyright 2026 Elsevier. (d) The PDOS for Pd 4d of Pd_3_BiC*_x_* and Pd_3_Bi. (e) The free energy profiles of the MBY reaction coordinate over Pd_3_BiC*_x_* and Pd_3_Bi. (d, e) Reproduced with permission from ref^58^. Copyright 2026 American Chemical Society.

Benefiting from the synergistic effects, the optimized catalyst achieves 95.6% alkynol conversion, 96.3% alkenol selectivity, and 80.6% alkenol FE at −1.1 V (*vs.* Ag/AgCl), along with a remarkable cathodic energy efficiency of 40 mol·h^–1^. Previous studies have revealed that the incorporation of the p-block metals into Pd catalysts induces robust p-d orbital hybridization, which modulates the shift of the Pd d-band center and modulates intermediate adsorption behavior.^164,165^ Notably, such metallic modulation can be further coupled with nonmetallic interstitial doping, which effectively suppresses subsurface H* formation and inhibits over-hydrogenation. Based on this dual-modulation strategy, Wang *et al.* embedded carbon atoms into PdBi intermetallic metallene (Pd_3_BiC_*x*_ ene) to overcome the inherent bottlenecks of low selectivity and unsatisfactory FE.^58^ Theoretical calculations verify that the constructed p-d-p orbital hybridization originating from C doping effectively tunes the electronic structure of Pd. This modulation optimizes the adsorption of alkynol substrates while weakening the binding affinity of alkenol products (Figure 15d). In addition, both the intermetallic structure and carbon doping also kinetically hinder overhydrogenation by eliminating subsurface hydrogen species and increasing the energy barrier for the overhydrogenation step (Figure 15e). Consequently, the Pd_3_BiC_*x*_ ene catalyst achieves excellent electrocatalytic performance, with 94.1% substrate conversion, 95.3% alkene selectivity, and 90.4% FE at −0.3 V vs. RHE.

### 4.4 Novel Applications in Sustainable Synthesis via Electrocatalytic Alkyne Semihydrogenation

Distinct from conventional thermal catalysis, electrocatalytic hydrogenation relies on potential regulation, interfacial electron transfer, and bipolar reaction coupling, giving rise to a series of unique advanced applications that are difficult to achieve with traditional thermal systems. Accordingly, this section summarizes the recent progress and emerging unique applications of advanced electrocatalytic alkyne hydrogenation systems.

(1) Electrocatalytic deuteration system: The C–D bond in deuterated drugs is inherently more stable than the C–H bond, which reduces metabolic clearance rates, extends drug half-life, and inhibits toxic metabolite formation, granting deuteration prominent advantages for precision medicine. Beyond pharmaceutical applications, deuterated reagents are widely applied for labeling and tracing molecular transformation and transfer processes in chemical reactions or biological systems, and support cutting-edge deuterium metabolic imaging studies. The integration of electrocatalysis into deuterium chemistry allows researchers to tune the yield and selectivity of different deuterated products by adjusting the supply rate of D*, suppressing the competing deuterium evolution side reaction, and optimizing the kinetic matching between substrates and catalytic active sites. Additionally, precise control over the applied potential enables selective deuteration for a wide range of substrates, covering electron-donating and electron-withdrawing compounds, alkenes with other reducible functional groups, as well as complex natural products and pharmaceutical molecules (Table 3).^84,85,153^

**Table 3:**
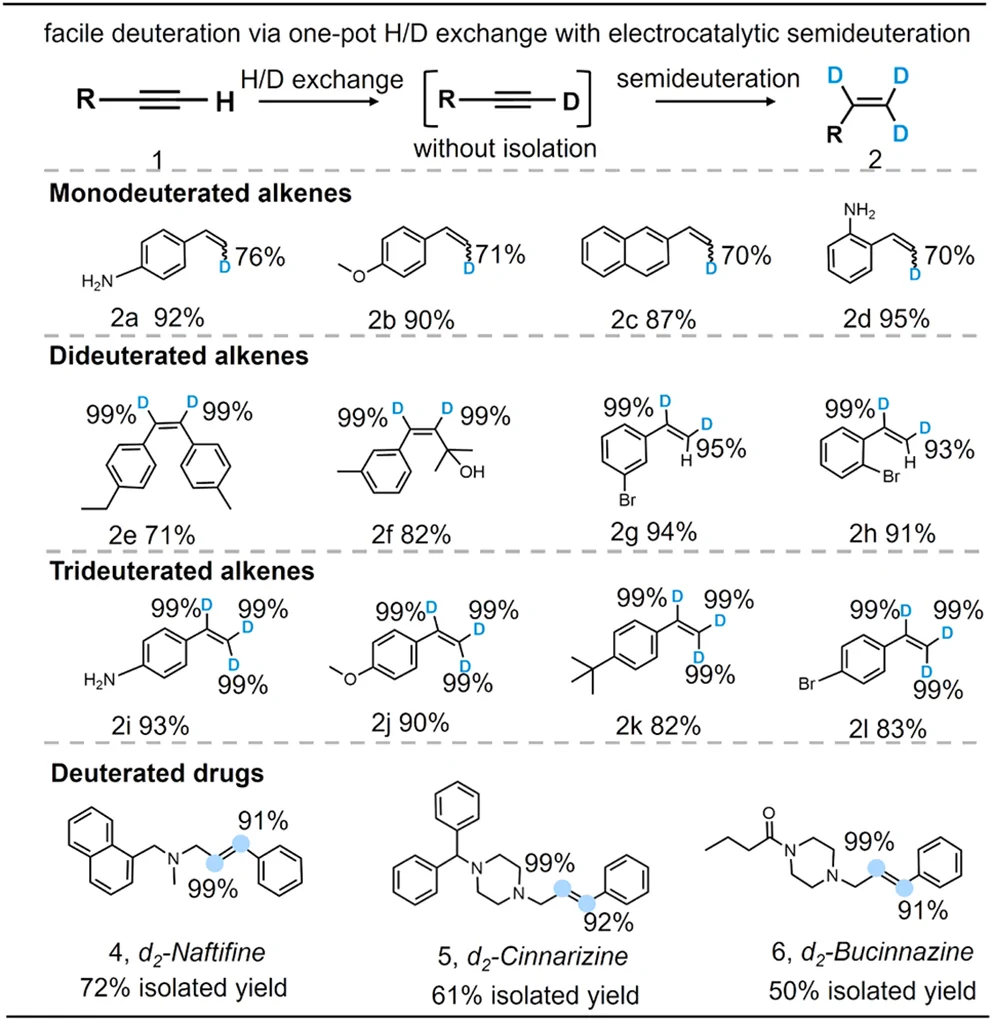
Mono-, Di-, Tri- and Complex Deuteration Products for the Electrocatalytic Transfer Semideuteration of Alkynes with D_2_O over Cathode Catalysts

Paired electrolysis systems: Paired electrolysis is a unique configuration of electrocatalytic systems that relies on dual-chamber electrolytic cells equipped with proton and anion exchange membranes. In the parallel mode, the cathode and anode process different reactants to yield distinct high-value products. For instance, Pan’s research team replaced the high-overpotential oxygen evolution reaction with glycerol oxidation for formic acid synthesis, while conducting electrocatalytic hydrogenation of 2-butylene-1,4-diol at the cathode.^166^ This integrated system reduced energy consumption by 40% at 200 mA cm^–2^, verifying its superior energy efficiency and economic benefits. Moreover, water-driven electrocatalytic hydrogenation enables the one-pot synthesis of complex organic molecules from simple substrates without intermediate separation. Yu’s team developed a cathode-anode tandem strategy for direct electrosynthesis of C–C–N compounds. CO_2_ is reduced to ethanol for C–C bond formation, and nitrate is converted to ammonia at the cathode; the *in-situ* generated ethanol and ammonia further undergo anodic oxidative coupling to construct C–N bonds and produce acetamide.^167^ In addition, paired electrolysis includes divergent and convergent modes. The divergent mode employs identical reactants in both chambers to generate different oxidation and reduction products, while the convergent mode uses different substrates at the two electrodes to produce the same target product. Such flexible cathode–anode coupling strategies effectively lower overall energy input and significantly expand the synthetic diversity of electrocatalytic organic hydrogenation.
Self-powered electrocatalytic hydrogenation systems: The design of electricity-driven electrocatalytic hydrogenation systems enables the integration of high-value chemical synthesis and electricity generation in one device. For example, leveraging insights from sacrificial anode protection and basic battery electrochemistry, Zhang’s research team reconfigures the corrosion reaction as a macroscopic spontaneous corrosion cell, pairing anodic metal-organic framework (MOF) synthesis with cathodic HER and electrocatalytic hydrogenation.^168^ This coupled design accelerates anodic corrosion to promote MOF formation, while unlocking simultaneous synergy between metal-organic framework growth, high-value chemical manufacturing, and electricity generation. This newly developed electrocatalytic hydrogenation system improves the overall energy efficiency while producing high-value chemicals, and is expected to represent a transformative upgrade of paired electrolysis from an energy-intensive process to a net power-generating model.

## 5 Conclusions and Perspectives

Alkyne semihydrogenation is a core reaction for the production of bulk functional materials and fine chemicals, and thus occupies a vital position in the chemical industry. Pd-based catalysts are the most widely used for alkyne hydrogenation owing to their outstanding catalytic activity. Nevertheless, unmodified Pd metal tends to cause severe overhydrogenation side reactions, leading to the classic trade-off between high activity and poor alkene selectivity. To address this issue, various modification strategies have been developed, including alloying, organic ligand functionalization, and atomic-level dispersion of Pd. These approaches are designed to regulate the adsorption behavior of alkenes and improve product selectivity. However, these strategies still suffer from notable limitations such as complicated preparation procedures, reliance on expensive or toxic metals, and inevitable activity loss, which greatly hinder their large-scale practical application. In recent years, the modulation of Pd by nonmetallic elements has emerged as a promising and powerful strategy for catalyst design. Well-designed interstitial nonmetal-doped Pd catalysts have been demonstrated to deliver excellent catalytic activity and selectivity, and have become a major research hotspot in the field of selective hydrogenation. In this review, we systematically review the latest research advances in nonmetal-modified Pd catalyst systems and mainly elaborate on the intrinsic mechanisms by which nonmetallic dopants optimize the performance of Pd catalysts in both thermocatalytic and electrocatalytic alkyne semihydrogenation. Studies have shown that nonmetals act as ideal promoters via dual regulation of electronic and geometric effects. On one hand, the incorporation of nonmetallic atoms tunes the electronic properties of Pd, downshifts its d-band center, and weakens the adsorption strength of reactants and intermediates, thus balancing catalytic activity and selectivity. On the other hand, nonmetal doping reconstructs the geometric structure of Pd surfaces, achieving active site blocking and ensemble isolation that effectively suppress overhydrogenation. Overall, doping Pd with nonmetals such as B, C, N, P, and S opens up new opportunities for the development of high-performance catalysts toward alkyne semihydrogenation. Despite the remarkable progress achieved so far, there are still several critical issues restricting the practical applications of these nonmetal-doped Pd-based catalysts.

Development of synthetic methodologies for atomically precise nonmetal doping: Although the currently reported synthetic approaches can successfully fabricate nonmetal-doped Pd-based catalysts with superior catalytic performance, it remains difficult for these conventional methods to precisely control the crystal phase, surface morphology, atomic arrangement, and local coordination environment of active sites. Most nonmetal interstitial doping approaches rely on high-temperature thermal treatment, which readily induces undesirable effects, including uncontrolled crystallization with large-grain formation, uneven dopant distribution, nanoparticle sintering, and random phase transformation. This inevitably leads to nonuniform microstructures and inconsistent electronic properties across catalyst samples. Such poor controllability greatly hinders the establishment of an unambiguous structure-performance relationship and restricts the rational design of high-efficiency Pd-based semihydrogenation catalysts. Given the abundant compositional and structural diversity and unique physicochemical characteristics of nonmetal-doped Pd catalyst systems, the development of advanced, atomically precise synthetic strategies is urgently needed. Future efforts should prioritize mild synthesis routes, spatially confined synthesis, and stepwise phase engineering to precisely regulate dopant dosage, type, and lattice location, and facilitate selective formation of target interstitial phases. Innovation in precise doping and phase engineering methodologies will substantially promote the targeted regulation of active structures, facilitate the performance optimization of Pd-based hydrogenation catalysts, and further advance research in selective alkyne semihydrogenation and related catalytic conversion fields.
Machine learning-assisted high-throughput screening of nonmetal doping systems: Traditional experiment-driven catalyst design is time-consuming and labor-intensive, which greatly limits the exploration scope of diverse nonmetal-doped Pd phases and composite configurations. There is an urgent demand for efficient strategies to predict the optimal nonmetal doping dosage, identify the most favorable electronic structure of Pd active centers, and clarify the intrinsic correlation between d-band electronic modulation and hydrogenation performance. This will greatly expand the catalyst screening space and accelerate the discovery of high-efficiency hydrogenation catalysts, for which machine learning offers a powerful solution.^169,170^ Although dedicated machine-learning investigations targeting nonmetal-doped Pd catalysts for alkyne semihydrogenation remain scarce, analogous studies on alloy and intermetallic catalysts for alkyne semihydrogenation have demonstrated the ability of machine learning to construct interpretable catalytic descriptors and rapidly screen promising catalyst candidates.^169,170^ By integrating extensive data on catalyst structure, catalytic performance, and reaction mechanism, it is feasible to construct comprehensive databases and rational catalytic descriptors to guide targeted research on alkyne semihydrogenation. Future efforts should be devoted to simulating diverse nonmetal-doped Pd catalyst models and deeply exploring the atomic-level hydrogenation reaction mechanism.
In-depth elucidation of dynamic reaction mechanisms under working conditions: Nonmetal incorporation induces electronic and geometric modulation of the Pd subsurface and active sites, and these modulations are highly dynamic during continuous alkyne semihydrogenation. Various surface reaction intermediates and *in situ* evolved interstitial phases (Pd–C and Pd–H) are transient, making it difficult to accurately capture their structural evolution and spectroscopic information. As a result, obtaining systematic and in-depth mechanistic understanding of semihydrogenation remains a major challenge. To resolve these fundamental problems, advanced *in situ* or operando characterization techniques and multi-scale theoretical simulations need to be further developed. Promising characterization tools include operando synchrotron-based XANES/EXAFS, grazing-incidence X-ray diffraction (GIXRD), near-ambient pressure XPS (NAP-XPS), operando CO–DRIFTS, and *in situ* attenuated total reflection Fourier-transform infrared spectroscopy (ATR-FTIR).^171–173^ These techniques enable real-time tracking of lattice distortion, electronic state evaluation, dopant stability, as well as adsorption/desorption behavior of alkynes and alkenes under realistic conditions.
Exploration of scalable synthetic routes and sustainable industrial implementation: It is of great importance to translate the promising performance of laboratory catalysts into practical industrial applications. Current research on nonmetal-modified Pd catalysts for alkyne semihydrogenation is dominated by laboratory-scale synthesis and performance evaluation. The large-scale and controllable preparation of stable nonmetal-doped Pd-based nanocatalysts still faces considerable technical hurdles, which hinder their industrial application. Exploring feasible industrial production pathways and evaluating catalyst performance, long-term stability, economic viability, and environmental compatibility under realistic industrial conditions will greatly facilitate the commercial application of such catalysts. Meanwhile, the industrial scale-up of electrocatalytic alkyne semihydrogenation is still in its initial stage, and appropriate reaction equipment and standardized operating procedures remain to be developed for future industrial implementation.
